# Anti-Wrinkling Effect of 3,4,5-tri-*O*-caffeoylquinic Acid from the Roots of *Nymphoides peltata* through MAPK/AP-1, NF-κB, and Nrf2 Signaling in UVB-Irradiated HaCaT Cells

**DOI:** 10.3390/antiox12101899

**Published:** 2023-10-23

**Authors:** Tae-Young Kim, No-June Park, Beom-Geun Jo, Bum Soo Lee, Min-Ji Keem, Taek-Hwan Kwon, Ki Hyun Kim, Su-Nam Kim, Min Hye Yang

**Affiliations:** 1 Department of Pharmacy, College of Pharmacy and Research Institute for Drug Development, Pusan National University, Busan 46241, Republic of Korea; taeyour@pusan.ac.kr (T.-Y.K.); bg_jo@pusan.ac.kr (B.-G.J.); mj_keem@pusan.ac.kr (M.-J.K.); thkwon@pusan.ac.kr (T.-H.K.); 2 Natural Products Research Institute, Korea Institute of Science and Technology, Gangneung 25451, Republic of Korea; h20508@kist.re.kr; 3 Division of Bio-Medical Science and Technology, KIST School, University of Science and Technology, Seoul 02792, Republic of Korea; 4 School of Pharmacy, Sungkyunkwan University, Suwon 16419, Republic of Korea; kosboybs@skku.edu

**Keywords:** *Nymphoides peltata*, 3,4,5-tri-*O*-caffeoylquinic acid (TCQA), anti-wrinkle, antioxidant, Nrf2, NF-κB, MAPK, AP-1, MMP-1

## Abstract

*Nymphoides peltata* has been widely used pharmacologically in traditional Chinese medicine to treat heat strangury and polyuria. The aim of this study was to isolate the bioactive components from *N. peltata* and evaluate their potential use as antioxidant and anti-wrinkle agents. Phytochemical investigation of the methanolic extract of *N. peltata* roots led to the isolation of 15 compounds (**1**–**15**), which were structurally determined as α-spinasterol (**1**), 3-*O*-*β*-D-glucopyranosyl-oleanolic acid 28-*O*-*β*-D-glucuronopyranoside (**2**), 4-hydroxybenzoic acid (**3**), protocatechuic acid (**4**), vanillic acid (**5**), *p*-coumaric acid (**6**), caffeic acid (**7**), ferulic acid (**8**), neochlorogenic acid (neo-CQA) (**9**), chlorogenic acid (CQA) (**10**), cryptochlorogenic acid (crypto-CQA) (**11**), isochlorogenic acid B (3,4-DCQA) (**12**), isochlorogenic acid A (3,5-DCQA) (**13**), isochlorogenic acid C (4,5-DCQA) (**14**), and 3,4,5-tri-*O*-caffeoylquinic acid (TCQA) (**15**). Of these 15 compounds, compound **2** was a new oleanane saponin, the chemical structure of which was characterized by 1D and 2D nuclear magnetic resonance (NMR) spectroscopic data and high-resolution electrospray ionization mass spectrometry (HRESIMS), as well as chemical reaction. Biological evaluation of the isolated compounds revealed that 3,4,5-tri-*O*-caffeoylquinic acid (TCQA) significantly improved Nrf2 levels in an Nrf2–ARE reporter HaCaT cell screening assay. TCQA was found to potently inhibit the Nrf2/HO-1 pathway and to possess strong anti-wrinkle activity by modulating the MAPK/NF-κB/AP-1 signaling pathway and thus inhibiting MMP-1 synthesis in HaCaT cells exposed to UVB. Our results suggest that TCQA isolated from *N. peltata* might be useful for developing effective antioxidant and anti-wrinkle agents.

## 1. Introduction

The skin is the largest body organ, and it protects internal organs from physical harm and chemical irritants, and plays an important role in maintaining skin homeostasis [1]. However, complex interactions between multiple intrinsic and extrinsic factors cause skin aging [2]. Photoaging due to continuous ultraviolet (UV) exposure is a major contributor to skin aging and accounts for more than 80% of facial aging [3]. In particular, UVB irradiation can penetrate the epidermis and dermis and induce oxidative stress by generating reactive oxygen species (ROS) within cells and tissues, which can lead to skin inflammation, aging, and skin cancer [4]. Nuclear factor erythroid-2-related factor 2 (Nrf2) is activated by oxidative stress and regulates antioxidant activity and cellular redox levels by neutralizing ROS and reactive electrophiles [5,6,7,8]. In other words, when exposed to oxidative stress, Nrf2 dissociates from Keap1 and binds to antioxidant response element (ARE) to induce the expression of antioxidant-related enzymes, such as heme oxygenase-1 (HO-1) and NAD(P)H: quinone oxidoreductase 1 (NQO1), and thereby inhibits ROS-induced oxidative damage and skin aging [9,10].

Photoaging is characterized by the activation of key transcription factors, including mitogen-activated protein kinase (MAPK), nuclear factor-κB (NF-κB), and activator protein-1 (AP-1) [11]. These factors promote collagen degradation in skin tissue by inducing matrix metalloproteinase-1 (MMP-1), which leads to an abnormal skin structure [12,13]. MAPKs are phosphorylated and activated by ROS and oxidative stress, and they enter the nucleus and upregulate the transcription factors AP-1 and NF-κB [4,14]. AP-1 increases the expression of MMPs, including MMP-1, MMP-3, and MMP-9, which degrade the extracellular matrix (ECM) and basement membrane components [15,16,17,18]. In particular, MMP-1 is a collagen-degrading protease that promotes the degradation of procollagen type-I, a crucial constituent of ECM; thus, it contributes to skin aging and wrinkle formation [19,20]. NF-κB is another transcription factor that increases MMP-1 levels in the dermis and is activated by UVB-generated ROS [21]. Furthermore, activation of the NF-κB pathway results in the synthesis of proinflammatory enzymes and cytokines, leading to inflammation and tissue damage, enhanced MMP-1 production, and skin aging [22,23,24]. Therefore, agents with antioxidant properties that promote Nrf2 activation and inhibit the MAPK/NF-κB/AP-1 signaling pathway are considered potential anti-photoaging and anti-wrinkle agents. For this reason, research is being actively conducted to identify phytochemicals with antioxidant, anti-aging, and anti-wrinkle effects [25,26].

The genus *Nymphoides* is one of the five genera of the family Menyanthaceae and contains approximately fifty species worldwide [27]. Three species of this genus, that is, *Nymphoides coreana* (H. Lev.) H. Hara, *Nymphoides indica* (L.) Kuntze, and *Nymphoides peltata* (S. G. Gmel.) Kuntze, are endemic in Korea [28]. *Nymphoides peltata* (syn. *Limnanthemum peltatum*, *Menyanthes nymphoides*), known as Xing-Cai in China and Asaza in Japan, is a perennial aquatic plant that is widely distributed in temperate and subtropical regions of Eurasia, including Korea, Japan, and China [29]. In the Compendium of Materia Medica (Ben Cao Gang Mu), a compendium of traditional Chinese medicines, *Nymphoides* are mainly used to treat heat strangury and polyuria. Pharmacologically, *N. peltata* extracts have been reported to inhibit platelet-activating factor (PAF) and N-formyl methionyl-leucyl-phenylalanine (fMLP), which are both potent pro-inflammatory factors [30]. In our recent study, we found that a 95% EtOH extract of *N. peltata* roots ameliorated atopic dermatitis and activated the Nrf2/HO-1 signaling pathway [31]. As part of ongoing research for *N. peltata*, phytochemical investigation of the MeOH extract of *N. peltata* roots was conducted, which led to the isolation of 15 compounds (**1**–**15**) including a new oleanane saponin (**2**). The structures of the isolated compounds were determined by nuclear magnetic resonance (NMR) spectroscopic data and LC–MS analysis. Herein, we describe the isolation and structural determination of compounds **1**–**15,** evaluating their antioxidant, anti-photoaging, and anti-wrinkle effects in UVB-irradiated HaCaT cells.

## 2. Materials and Methods

### 2.1. General

Column chromatography was conducted using Silica gel 60 (70–230 mesh, Merck, Darmstadt, Germany) and Sephadex LH-20 (25–100 μm; Pharmacia, Stockholm, Sweden). Thin-layer chromatography (TLC) analysis was performed on silica gel 60 F254 Art. 5715 plates (Merck, Germany). High-performance liquid chromatography (HPLC) preparative analysis was performed using a Gilson HPLC system equipped with two pumps (305 master pump, 307 slave pump) and a mixer (811C dynamic mixer), and a Shimadzu HPLC system with a UV/vis detector (SPD-20A), pump (LC-20AT), and system controller (CBM-20A). HPLC was conducted using a Watchers 120 ODS-BP column (S-10 μm, 150 mm × 10 mm, Isu Industry Corp., Seoul, Republic of Korea). High-resolution electrospray ionization mass spectrometry (HR-ESI-MS) was conducted using an Agilent 6530 Accurate-Mass Quadrupole Time-of-Flight (Q-TOF) LC/MS system, and a 6545 Accurate-Mass Q-TOF LC/MS (Agilent Technologies, Santa Clara, CA, USA). Nuclear magnetic resonance (NMR) spectra were recorded using JEOL 400 MHz (JNM-ECZ400S, JEOL, Tokyo, Japan), Bruker 500 MHz (Bruker, Billerica, MA, USA), and Agilent Technologies 600 MHz (Santa Clara, CA, USA) instruments. DMSO-*d*_6_ and chloroform-*d* (Cambridge Isotope Laboratories, Andover, MA, USA) were used as solvents for NMR studies.

### 2.2. Plant Material

*N. peltata* roots were collected at the Hantaek Botanical Garden Foundation (Yongin-si, Gyeonggi-do, Republic of Korea) and authenticated by Dr. Jung Hwa Kang. Voucher specimens (PNU-0040) were deposited at the Medicinal Herb Garden, Pusan National University.

### 2.3. Isolation of Compounds from N. peltata Extract

Dried roots of *N. peltata* (2.8 kg) were first ground to a powder, soaked in 100% methanol (MeOH), ultrasonicated for 90 min at room temperature twice, and immediately freeze-dried. The yield of MeOH extract obtained was 15.25% (427 g). This extract was then suspended in 2 L of H_2_O and sequentially extracted with *n*-hexane (Hex), ethyl acetate (EA), and *n*-butanol (*n*-BuOH) to obtain three fractions: NPH (80.3 g), NPE (24 g), and NPB (108 g), respectively.

The NPH fraction was subjected to silica gel column chromatography using Hex:EA (8:1 → 100% EA gradient) as the eluant to obtain 12 fractions. The NPH-6 (17.8996 g) subfraction was subjected to silica gel column chromatography using Hex:EA (5:1 → 100% EA gradient) as the eluant to obtain 9 fractions. The NPH-6-4 (2.1 g) subfraction was recrystallized using 100% methanol (MeOH) to obtain pure compound **1** (21.3 mg).

The NPE fraction was subjected to silica gel column chromatography using EA:MeOH (10:1 → 100% MeOH gradient) as the eluant to obtain 7 fractions. The NPE-1 (463.2 mg) subfraction was subjected to silica gel column chromatography using EA:MeOH (20:1 → 100% MeOH gradient) as the eluant to obtain 6 fractions. The NPE-1-4 (10.1 mg) subfraction was subjected to semi-preparative TLC using a methylene chloride (CH_2_Cl_2_):MeOH = 20:1 mobile phase to obtain 6 fractions. The NPE-1-4-6 (0.5 mg) subfraction was subjected to the Shimadzu prep HPLC system (UV wavelengths 250 and 330 nm; flow rate 2 mL/min) using an ODS column using 0.1% formic acid in acetonitrile (ACN):0.1% formic acid in H_2_O (25:75 isocratic) as the eluant to obtain pure compound **3** (retention time (*t*_R_) 19 min, 0.4 mg). The NPE-2 (1.025 g) fraction was subjected to silica gel column chromatography using chloroform (CHCl_3_):MeOH (20:1 → 100% MeOH gradient) as the eluant to obtain 9 fractions. The NPE-2-2,3 (73.7 mg) subfraction was subjected to the Shimadzu prep HPLC system (UV wavelengths 250 and 330 nm; flow rate 2 mL/min) using an ODS column and 0.1% formic acid in ACN:0.1% formic acid in H_2_O (27:73 isocratic) as the eluant to obtain pure compounds **5** (*t*_R_ 13 min, 2.2 mg) and **8** (*t*_R_ 19 min, 2.3 mg). The NPE-2-9 (206 mg) fraction was subjected to Sephadex LH-20 column chromatography using 100% MeOH as the eluant to obtain 3 fractions. The NPE-2-9-1 (115.7 mg) subfraction was subjected to Sephadex LH-20 column chromatography using 100% MeOH as the eluant to obtain 5 fractions. The NPE-2-9-1-4 (18.1 mg) subfraction was subjected to Shimadzu prep HPLC (UV wavelengths 250 and 330 nm; flow rate 2 mL/min) using an ODS column and 0.1% formic acid in ACN:0.1% formic acid in H_2_O (18:82 isocratic) as the eluant to obtain pure compounds **4** (*t*_R_ 13 min, 0.5 mg), **7** (*t*_R_ 22.2 min, 4.2 mg), and **6** (*t*_R_ 39 min, 1.4 mg). The NPE-6 (5.9752 g) subfraction was subjected to Gilson prep HPLC (UV wavelength 250 nm; flow rate 2 mL/min) using an ODS column and MeOH:H_2_O (10:90 → 100% MeOH gradient) as the eluant to obtain 4 fractions. The NPE-6-3 (397.1 mg) subfraction was subjected to Shimadzu prep HPLC (UV wavelengths 250 and 330 nm; flow rate 2 mL/min) using an ODS column and 0.1% formic acid in ACN:0.1% formic acid in H_2_O (21:79 isocratic) as the eluant to obtain 7 fractions. The NPE-6-3-7 (147.4 mg) subfraction was subjected to the Shimadzu prep HPLC system (UV wavelengths 250 and 330 nm; flow rate 2 mL/min) using an ODS column and 0.1% formic acid in ACN:0.1% formic acid in H_2_O (30:70 isocratic) as the eluant to obtain 4 fractions. The NPE-6-3-7-4 (7.6 mg) subfraction was subjected to the Shimadzu prep HPLC system (UV wavelengths 250 and 330 nm; flow rate 2 mL/min) using an ODS column and 0.1% formic acid in ACN:0.1% formic acid in H_2_O (20:80 isocratic) as the eluant to obtain pure compound **15** (*t*_R_ 48 min, 4.2 mg). The NPE-7 (1.893 g) subfraction was subjected to Shimadzu prep HPLC (UV wavelengths 250 and 330 nm; flow rate 2 mL/min) using an ODS column and 0.1% formic acid in ACN:0.1% formic acid in H_2_O (20:80 isocratic) as the eluant to obtain pure compounds **12** (*t*_R_ 38 min, 13.2 mg), **13** (*t*_R_ 48 min, 24.3 mg) and **14** (*t*_R_ 60 min, 10.8 mg).

The NPB fraction was subjected to silica gel column chromatography using EA:MeOH (20:1 → 100% MeOH) as the eluant to obtain 5 fractions. The NPB-4 (28.7062 g) subfraction was subjected to silica gel column chromatography using Hex:EA (10:1 → 100% EA → 100% MeOH) as the eluant to obtain 5 fractions. The NPB-4-5 (19.4128 g) subfraction was subjected to silica gel column chromatography using CHCl_3_:MeOH (20:1 → 100% MeOH) as the eluant to obtain 7 fractions. The NPB-4-5-7 (8.5607 g) subfraction was subjected to Gilson prep HPLC (UV wavelength at 250 nm; flow rate 2 mL/min) using an ODS column and MeOH:H_2_O (10:90 → 100% MeOH gradient) as the eluant to obtain 6 fractions. The NPB-4-5-7-1 (200.8 mg) subfraction was subjected to the Shimadzu prep HPLC system (UV wavelengths 250 and 330 nm; flow rate 2 mL/min) using an ODS column and 0.1% formic acid in ACN:0.1% formic acid in H_2_O (10:90 isocratic) as the eluant to obtain pure compounds **9** (*t*_R_ 21 min, 7.8 mg), **10** (*t*_R_ 38 min, 15.4 mg), and **11** (*t*_R_ 46 min, 9.6 mg). The NPB-4-5-7-5 (512.9 mg) subfraction was recrystallized using 100% MeOH to obtain pure compound **2** (258.1 mg).

#### 3-*O*-*β*-D-Glucopyranosyl-Oleanolic Acid 28-*O*-*β*-D-Glucuronopyranoside (**2**)

White amorphous powder; ${\left[\alpha \right]}_{D}^{25}$ + 15.9 (c 0.2, MeOH); UV (MeOH) *λ*_max_ (log ε) 217 (3.8) nm; IR (neat) v_max_: 3450, 1742, 1725, 1075 cm^−1^; ^1^H (400 MHz) and ^13^C (100 MHz) NMR data (Table 1); HR-ESI-MS (positive ion mode) *m*/*z* 817.4332 [M + Na]^+^ (calcd. for C_42_H_66_NaO_14_, 817.4350).

### 2.4. Cell Culture and UVB Irradiation

HaCaT cells (a spontaneously immortalized human keratinocyte cell line) were purchased from N.E. Fusenig (Deutsches Krebsforschungszentrum, Heidelberg, Germany) and cultured in Dulbecco’s modified Eagle’s medium (DMEM; HyClone, Logan, UT, USA) supplemented with 10% fetal bovine serum (FBS) and 100 units/mL of penicillin and 100 mg/mL streptomycin (HyClone) at 37 °C in a 5% CO_2_ humidified atmosphere. Seeded HaCaT cells were pretreated with various concentrations of TCQA (3,4,5-tri-*O*-caffeoylquinic acid, compound **15**) for one hour and then exposed to 15 mJ/cm^2^ of UVB using a 280–360 mm light source (G15T8E UV-B lamp; Tokyo, Japan). UV strength was measured using a photo/radiometer (HD2102.1; Delta OHM, Padoba, Italy). After UVB exposure, cells were treated with various concentrations of TCQA (**15**) in 1% FBS.

### 2.5. Cell Viability

Cell viabilities were determined using a CCK-8 kit (WST-8; Abcam, Cambridge, MA, USA). HaCaT cells seeded in 96-well plates (2 × 10^4^ cells/well) were pretreated with the indicated concentrations of TCQA (**15**) for 4 h, washed with PBS, and then exposed to UVB (15 mJ/cm^2^) and TCQA (**15**) for 3, 6, 12, or 24 h in 1% FBS. After UVB exposure, CCK-8 assay reagent was added to each well, and the cells were incubated for 30 min at 37 °C. Formazan absorbances were measured at 450 nm using a multifunctional plate reader (Tecan Infinite M1000 Microplate Reader, Tecan, Männedorf, Zürich, Switzerland), and compared to untreated cells.

### 2.6. ROS Measurement (DCFDA Assay)

Intracellular ROS formation was assessed using dichlorofluorescin diacetate (DCFDA) as the substrate. HaCaT cells were seeded at 2 × 10^4^ cells/well in black 96-well plates and, 24 h later, pretreated with the indicated concentrations of TCQA (**15**) for 4 h. Then, they were washed with PBS and exposed to UVB (15 mJ/cm^2^) and TCQA (**15**) for 3, 6, 12, or 24 h in 1% FBS. After UVB irradiation, cells were incubated with DCFDA (20 μM in 1% PBS) for 30 min. DCF concentrations in media caused by the ROS-induced oxidation of DCFDA were measured at excitation/emission wavelengths of 485/525 nm using a multifunctional plate reader (Tecan Infinite M1000 Microplate Reader, Tecan).

### 2.7. Luciferase Reporter Gene Assay

HaCaT cells were seeded into 24-well plates, cultured for 24 h, and co-transfected with ARE-Luc reporter plasmid (300 ng/well) and internal control plasmid pRL-SV-40 (5 ng/well) using the FuGENE^®^ 4K Transfection Reagent (Fugent LLC, Madison, WI, USA). Twenty-four hours after transfection, cells were treated with the indicated concentrations of TCQA (**15**) for an additional 24 h. Luciferase activities of cell lysates were then measured using the Dual-Luciferase^®^ Reporter Assay System according to the manufacturer’s instructions (Promega, Madison, WI, USA). To determine transfection efficiencies, luciferase activities were expressed as ratios of Renilla luciferase activity (SV40).

Transactivation of AP-1 or NF-κB was evaluated using pNF-κB-Luc plasmid or pAP-1-luc plasmid (300 ng/well; Stratagene, San Diego, CA, USA) with pRL-SV40 (5 ng/well) in HaCaT cells. Twenty-four hours after transfection, the cells were pretreated with a TCQA (**15**) for 1 h. The cells were then treated with the proinflammatory cytokine [NF-κB (TNF-α and IFN-γ 10 ng/mL), AP-1 (PMA 2 μM)] for 20 h. Transactivation of NF-κB or AP-1 was determined as described above.

### 2.8. Real-Time Quantitative PCR (qPCR)

HaCaT cells were seeded at 2 × 10^5^ cells/well in 12-well plates. Then, 24 h later, they were pretreated with the indicated concentrations of TCQA (**15**) for 4 h, washed with PBS, and treated with UVB (15 mJ/cm^2^) and TCQA (**15**) for 24 h when total RNA was isolated. This was carried out using an RNeasy mini kit (Qiagen, Hilden, Germany) and reverse transcribed using a RevertAid first-strand cDNA synthesis kit (Thermo Fisher Scientific, Waltham, MA, USA). Real-time PCR was conducted using QuantaStudio 6 pro (Thermo Fisher Scientific) using SYBR1 Green (Power SYBR Green PCR Master Mix; Applied Biosystems, Foster City, CA, USA). PCR settings were as follows: initial incubation for 2 min at 50 °C, denaturing for 10 min at 95 °C, followed by 40 cycles of PCR (15 s at 95 °C and 60 s at 60 °C). The primer sets used were MMP-1 (accession no. DQ399597), forward 5′-GCC CAG ATG TGG AGT GCC TG-3′ and reverse 5′-GTT TGC TCC CAG CGA GGG TT-3′; and GAPDH (accession no. NM_001357943), forward 5′-ACA CCC ACT CCT CCA CCT TT-3′ and reverse 5′-TGC TGT AGC CAA ATT CGT TG-3′. The qPCR data were analyzed using the QuantaStudio 6 pro System (Thermo Fisher Scientific). Transcript levels were normalized versus GAPDH.

### 2.9. Western Blot Analysis

Protein levels were measured by Western blot. Briefly, HaCaT cells were seeded at 5 × 10^5^ cells/well in 6-well plates. Then, 24 h later, they were pretreated with the indicated concentrations of TCQA (**15**) for 4 h, washed with PBS, and exposed to UVB (15 mJ/cm^2^). After 24 h, cells were lysed in appropriate amounts of RIPA buffer (Bioprince, Chuncheon, Republic of Korea) and Phosphatase Inhibitor Cocktail 2 (Sigma-Aldrich, St. Louis, MO, USA). Lysates were centrifuged at 13,000 rpm for 15 min at 4 °C, and supernatants were collected for further studies. Denatured protein lysates were separated by SDS-PAGE and transferred to PVDF membranes (Merck, Darmstadt, Germany). Blots were treated with primary antibodies against p-Nrf2 (1/1000; Invitrogen, Carlsbad, CA, USA), Nrf2 (1/1000; BioLegend, San Diego, CA, USA), HO-1 (1/1000; Abcam), p-extracellular signal-regulated kinase (ERK)1/2 (1/1000; Cell Signaling, Danvers, MA, USA), ERK1/2 (1/1000; Santa Cruz Biotechnology, Santa Cruz, CA, USA), p-c-Jun N-terminal kinase (JNK) (1/1000; Cell Signaling), JNK (1/1000; Santa Cruz Biotechnology), p-p38 (1/1000; Cell Signaling), p38 (1/1000; Santa Cruz Biotechnology), p-c-fos (1/1000; Cell Signaling), c-fos (1/1000; Santa Cruz Biotechnology), p-c-jun (1/1000; Santa Cruz Biotechnology), c-jun (1/1000; Santa Cruz Biotechnology), p-nuclear factor of kappa light polypeptide gene enhancer in B-cells inhibitor, alpha (IκBα) (1/1000; Cell Signaling), IκBα (1/1000; Cell Signaling), p-p65 (1/1000; Cell Signaling), p65 (1/1000; Cell Signaling), and glyceraldehyde-3-phosphate dehydrogenase (GAPDH) (1/3000; Cell Signaling). Then, they were treated with horseradish-peroxidase-conjugated secondary antibodies (rabbit; 1/3000; Cell Signaling, mouse; 1/3000, Santa Cruz Biotechnology) and visualized using an ECL kit (Thermo Fisher Scientific).

### 2.10. Determination of MMP-1 Secretions by ELISA

HaCaT cells were seeded at 2 × 10^5^ cells/well in 12-well plates. Then, 24 h later, they were pretreated with TCQA (**15**) (5, 10, or 20 μM) for 4 h, washed with PBS, and then exposed to UVB (15 mJ/cm^2^) and TCQA (**15**) overnight in 1% FBS medium. After exposure to UVB, culture supernatants were collected and centrifuged at 13,000 rpm for 5 min. Concentrations of matrix metalloproteinase-1 secreted into culture media were determined using human total MMP-1 enzyme-linked immunosorbent assay (ELISA) kits (Human Total MMP-1 DuoSet ELISA kit; R&D Systems, Minneapolis, MN, USA). Absorbances at 450 nm were read using a microplate reader (Tecan Infinite M1000 Microplate Reader, Tecan).

### 2.11. Statistical Analysis

The statistical analysis was performed using GraphPad Prism software v4.0 (GraphPad, La Jolla, CA, USA), and results are presented as the means ± SDs of 2 to 3 independent experiments. One-way analysis of variance (ANOVA) followed by Tukey’s multiple comparisons test for independent samples was used to determine the significances of intergroup differences, and statistical significance was accepted for *p*-values < 0.05: ^#^ *p* < 0.05, ^##^ *p* < 0.01, and ^###^ *p* < 0.001; * *p* < 0.05, ** *p* < 0.01, and *** *p* < 0.001.

## 3. Results

### 3.1. Isolation and Structural Identification of Compounds from N. peltata 

**Figure 1:**
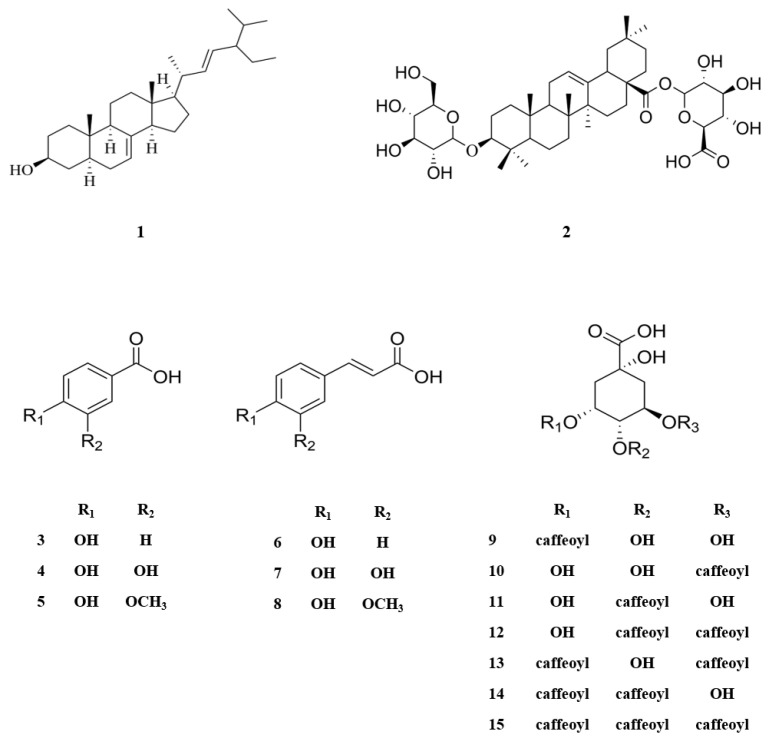
Chemical structures of compounds **1**–**15** isolated from *N. peltata*.

The MeOH extract of *N. peltata* roots was subjected to phytochemical investigation through liquid–liquid partitioning, column chromatography, and HPLC, leading to the isolation of one steroid derivative, one terpenoid saponin, six phenolic acid derivatives, and seven caffeoylquinic acid derivatives. The isolated compounds (**1–15**) were structurally identified as follows: α-spinasterol (**1**) (Figure S1) [32], 3-*O*-*β*-D-glucopyranosyl-oleanolic acid 28-*O*-*β*-D-glucuronopyranoside (**2**) (Figures S2–S6), 4-hydroxybenzoic acid (**3**) (Figure S7) [33], protocatechuic acid (**4**) (Figure S8) [34], vanillic acid (**5**) (Figure S9) [34], *p*-coumaric acid (**6**) (Figure S10) [35], caffeic acid (**7**) (Figure S11) [36], ferulic acid (**8**) (Figure S12) [37], neochlorogenic acid (neo-CQA) (**9**) (Figure S13) [38], chlorogenic acid (CQA) (**10**) (Figure S14) [38], cryptochlorogenic acid (crypto-CQA) (**11**) (Figure S15) [38], isochlorogenic acid B (3,4-DCQA) (**12**) (Figure S16) [39], isochlorogenic acid A (3,5-DCQA) (**13**) (Figure S17) [39], isochlorogenic acid C (4,5-DCQA) (**14**) (Figure S18) [39], and 3,4,5-tri-*O*-caffeoylquinic acid (TCQA) (Figure S19) (**15**) [40] (Figure 1). These identifications were based on their LC/MS data and comparison of their NMR spectroscopic data with those previously reported. A literature survey revealed that compound **2** is a new oleanane saponin, the chemical structure of which was characterized by 1D and 2D NMR spectroscopic data and high-resolution electrospray ionization mass spectrometry (HRESIMS), as well as chemical reaction.

**Figure 2:**
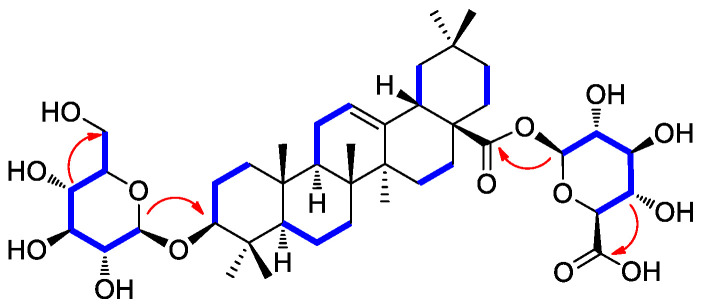
^1^H-^1^H COSY (blue lines) and key HMBC correlations (red arrows) of compound **2**.

Compound **2** was isolated as a white amorphous powder with a molecular formula of C_42_H_66_O_14_, as deduced from the molecular ion peak [M + Na]^+^ at *m*/*z* 817.4332 (calcd. for C_42_H_66_NaO_14_, 817.4350) in the positive-ion mode of HRESIMS. The ^1^H NMR data (Table 1) of compound **2** showed proton NMR resonances corresponding to seven methyl groups [*δ*_H_ 0.67 (3H, s), 0.74 (3H, s), 0.86 (3H, s), 0.86 (3H, s), 0.87 (3H, s), 0.96 (3H, s), and 1.07 (3H, s)], one oxymethine proton [*δ*_H_ 3.01 (1H, dd, *J* = 10.5, 3.5 Hz)], and one olefinic proton [*δ*_H_ 5.16 (1H, br s)]. This was in addition to proton signals for sugar units, including two anomeric protons at *δ*_H_ 4.13 (1H, d, *J* = 7.5 Hz) and *δ*_H_ 5.23 (1H, d, *J* = 8.0 Hz), and other oxygenated protons at *δ*_H_ 3.43 (1H, dd, *J* = 11.0, 5.5 Hz), *δ*_H_ 3.61 (1H, dd, *J* = 10.0, 5.0 Hz), and *δ*_H_ 3.28–2.95. The ^13^C NMR data (Table 1) of compound **2** showed 42 carbon resonances, including seven methyl groups (*δ*_C_ 15.7, 17.0, 17.1, 23.8, 26.0, 28.1, and 33.2), one highly deshielded oxygenated methine (*δ*_C_ 88.4), two olefinic methines (*δ*_C_ 122.2 and 143.9), and one carboxyl carbon (*δ*_C_ 175.7) for an aglycone part of oleanolic acid. Among the remaining carbon signals attributable to sugar units, anomeric carbon signals at *δ*_C_ 94.6 and *δ*_C_ 101.7, and characteristic oxygenated carbon at *δ*_C_ 61.1 and carboxyl carbon at *δ* 174.3 indicated the presence of glucose and glucuronic acid, respectively [41,42]. Overall, ^1^H and ^13^C NMR data and mass spectral data implied that compound **2** is an oleanane-type triterpenoid saponin, and the NMR data were revealed to be very similar to those of chikusetsusaponin IVa [41,42]. However, there were some noticeable differences in the ^13^C NMR chemical shifts observed for the sugar groups, which suggested that compound **2** may have the different linkages of glucose and glucuronic acid from chikusetsusaponin IVa. The accurate structure of compound **2** was confirmed by ^1^H−^1^H COSY, HSQC, and HMBC experiments. The ^1^H−^1^H COSY correlations starting at H-1′ via H-2′/H-3′/H-4′/H-5′ and ending at H2–6′ as well as the HMBC correlations of H-1′/C-3 (*δ*_C_ 88.4) and H-4′/C-6′ (*δ*_C_ 61.1) determined the linkage of glucose at C-3 (Figure 2). In addition, the key HMBC correlations of H-1″/C-28 (*δ*_C_ 175.7) and H-4″/C-6″ (*δ*_C_ 174.3) and ^1^H−^1^H COSY correlations of H-1″/H-2″/H-3″/H-4″/H-5″ confirmed the glucuronic acid moiety connected at C-28 (Figure 2). The completed gross structure of compound **2** was finally established via cross-peaks in the HMBC and ^1^H−^1^H COSY spectra (Figure 2). For the absolute configuration of sugar units, sugar moieties of compound **2** were gained through acid hydrolysis and then determined by LC/MS–UV as described in the previous paper [42], which verified the absolute configuration for both glucose and glucuronic acid as the D-form. The anomeric coupling constants (*J* = 7.5 Hz for glucose and *J* = 8.0 Hz for glucuronic acid) were indicative of the β-form, confirming the sugar units as β-D-glucose and β-D-glucuronic acid, respectively [41,42]. Finally, the aglycone, derived from acid hydrolysis, was identified as oleanolic acid by comparison of the optical rotation and NMR data [41,42]. Accordingly, the chemical structure of compound **2** was elucidated as 3-*O*-*β*-D-glucopyranosyl-oleanolic acid 28-*O*-*β*-D-glucuronopyranoside, and named peltatasaponin A.

### 3.2. Effects of Compounds Isolated from N. peltata on Nrf2–ARE Luciferase Activity in HaCaT Cells

**Figure 3:**
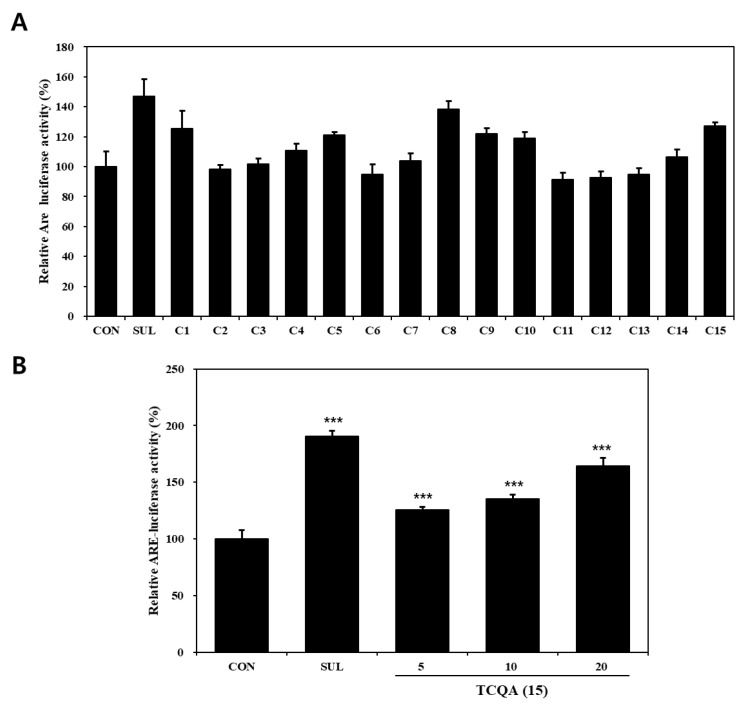
Nrf2–ARE luciferase activities of compounds isolated from *N. peltata*. Transfected HaCaT cells were treated with (**A**) each of the fifteen compounds isolated *N. peltata* (10 μM) or (**B**) TCQA (5 μM, 10 μM, and 20 μM), and sulforaphane (2 μM) for 24 h. Results are presented as means ± SDs (*n* = 3). *** *p* < 0.001 versus CON group; CON: untreated control group; SUL: sulforaphane-treated group; TCQA: TCQA-treated group.

The effects of compounds (**1**–**15**) on the Nrf2–ARE pathway, as determined using Nrf2–ARE reporter, in HaCaT cells are shown in Figure 3A. Regarding activation of the Nrf2–ARE pathway, compounds **1** (125%), **8** (138%), **10** (119%), and **15** (127%) significantly increased Nrf2–ARE luciferase activity compared to the untreated control (CON) group (100%). Out of the four, compounds **1**, **8**, and **10** have already been reported to have potential use as skin anti-aging treatments due to their ability to activate Nrf2 [43,44,45]. TCQA (**15**) treatment of Nrf2–ARE reporter HaCaT cells resulted in a significant concentration-dependent increase in Nrf2 activity, that is, it enhanced activity by 25% at 5 μM, 35% at 10 μM, and 54% at 20 μM, compared to the CON group (Figure 3B).

### 3.3. Effects of TCQA (15) on Antioxidant Activity in HaCaT Cells 

**Figure 4:**
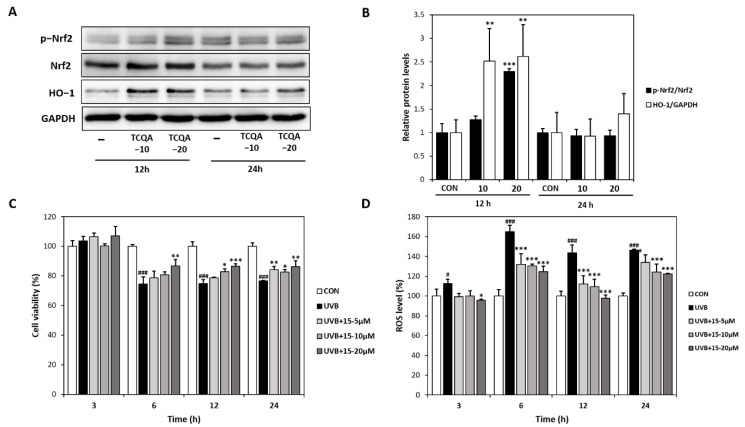
Effects of TCQA on Nrf2 expression, UVB-induced cell viability, and ROS levels in HaCaT cells. (**A**,**B**) pNrf2, Nrf2, and HO-1 protein expressions were analyzed by Western blot (normalized vs. GAPDH). (**C**) Cell viabilities were determined using a CCK-8 assay, and (**D**) ROS levels were assessed using a DCFDA assay. HaCaT cells were treated with TCQA at 5, 10, or 20 μM and cultured in the absence or presence of UVB for 3, 6, 12, and 24 h. Data are expressed as means ± SDs (*n* = 2 to 3). ^#^ *p* < 0.05 and ^###^ *p* < 0.001 versus CON group; * *p* < 0.05, ** *p* < 0.01, *** *p* < 0.001, versus CON or UVB-treated group (UVB). CON: untreated control group; UVB: UVB-treated group; TCQA: TCQA-treated group.

Treatment of HaCaT cells with TCQA for 12 h dose-dependently increased Nrf2 and HO-1 protein expressions by 1.3- and 2.3-fold at 10 μM and by 2.5- and 2.6-fold at 20 μM, respectively, compared to the CON group (Figure 4A,B). The CCK-8 assay showed that cultured HaCaT cells exhibited high ROS levels after exposure to UVB (6, 12, or 24 h). However, treatment with TCQA dose-dependently inhibited apoptosis and maintained cell viability for 24 h (Figure 4C). Furthermore, DCFDA analysis showed ROS levels were concentration-dependently reduced by TCQA at 5, 10, or 20 μM, compared to the UVB-treated (UVB) group. ROS levels were reduced by 20% (5 μM), 21% (10 μM), and 25% (20 μM) after 6 h of TCQA treatment and by 21% (5 μM), 24% (10 μM), and 31% (20 μM) after 12 h of TCQA treatment (Figure 4D).

### 3.4. Effects of TCQA on MAPK-, AP-1-, and NF-κB-Related Transcription Factors in HaCaT Cells Exposed to UVB

**Figure 5:**
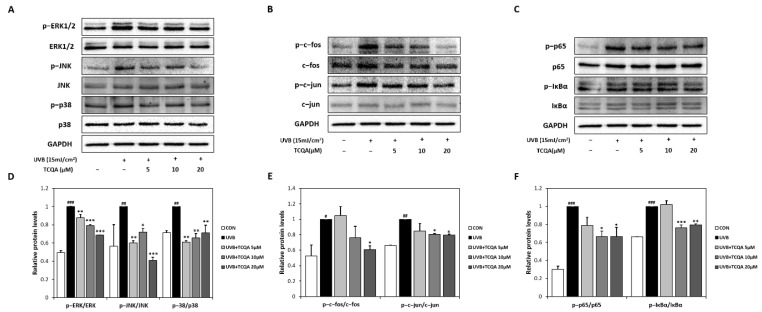
Effect of TCQA on ERK, p38 and JNK/MAPK, c-fos and c-jun/AP-1, and p65 and IκBα/NF-κB transcription activities. After treating cells with the indicated concentrations of TCQA for 24 h. (**A**,**D**) ERK, p38, and JNK; (**B**,**E**), c-fos and c-jun; and (**C**,**F**) p65 and IκBα. The expressions of subunits gene were analyzed by Western blot (normalized vs. GAPDH) in HaCaT cells exposed to UVB. Data are expressed as means ± SDs (*n* = 2 to 3). ^#^ *p* < 0.05, ^##^ *p* < 0.01 and ^###^ *p* < 0.001, versus CON group; * *p* < 0.05, ** *p* < 0.01, and *** *p* < 0.001 versus UVB group. CON: untreated control group; UVB: UVB-treated group; TCQA: TCQA-treated group.

UVB exposure significantly increased the expression of MAPK-related transcription factors, such as p-ERK/ERK, p-JNK/JNK, and p-p38/p38. Treatment with TCQA significantly inhibited the expression levels of p-ERK/ERK (10% at 5 μM, 20% at 10 μM, and 30% at 20 μM), p-JNK/JNK (40% at 5 μM, 30% at 10 μM, and 60% at 20 μM), and p-p38/p38 (40% at 5 μM, 34% at 10 μM, and 30% at 20 μM), compared to the UVB group (Figure 5A,D). TCQA also decreased the expressions of AP-1-related transcription factors, such as p-c-fos/c-fos and p-c-jun/c-jun, in HaCaT cells exposed to UVB. Significant reductions were observed after treatment with 20 μM of TCQA, such as a 40% decrease in p-c-fos/c-fos expression and an 80% decrease in the p-c-jun/c-jun expression (Figure 5B,E). Furthermore, TCQA treatment dose-dependently inhibited the expressions of NF-κB-related transcription factors, such as p-p65/p65 and p-IκBα/IκBα. Significant inhibition was observed in the TCQA 10 μM group, with reductions of 33% and 25% in p-p65/p65 and p-IκBα/IκBα, respectively, compared to the UVB group (Figure 5C,F).

### 3.5. Effects of TCQA on AP-1 Activity in PMA-Induced HaCaT Cells and NF-κb Activity in TNF-α/IFN-γ-Induced HaCaT Cells

**Figure 6:**
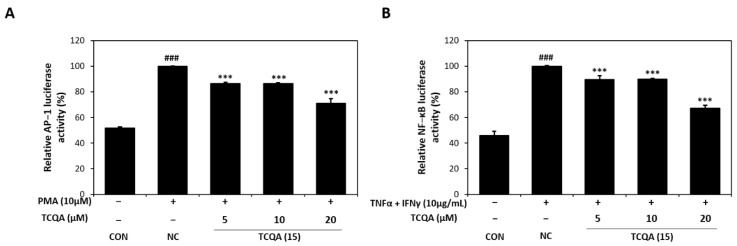
Effect of TCQA on AP-1 and NF-κB luciferase activities. (**A**) Transactivation of AP-1 or (**B**) NF-κB was evaluated using luciferase reporter gene assay in PMA or TNF-α/IFN-γ-induced HaCaT cells. Data are expressed as mean ± SD (*n* = 3). ^###^ *p* < 0.001, compared with the CON group; *** *p* < 0.001, versus NC group. CON: untreated control group; NC: PMA or TNF-α and IFN-γ-treated group; TCQA: TCQA-treated group.

As id shown in Figure 6A, AP-1 luciferase activity increased to 100% in PMA (10 μM)-induced HaCaT cells. On the other hand, AP-1 activity significantly decreased to 86%, 86%, and 71% in the 5, 10, and 20 μM TCQA groups, respectively, vs. the NC group. In addition, exposure to TNF-α/IFN-γ increased NF-κB luciferase activity to approximately 100% of the CON group (46%). However, TCQA at 5, 10, and 20 μM significantly reduced the activation of NF-κB by 12%, 11%, and 33%, respectively (Figure 6B).

### 3.6. Effects of TCQA on MMP-1 Expression in HaCaT Cells Exposed to UVB

**Figure 7:**
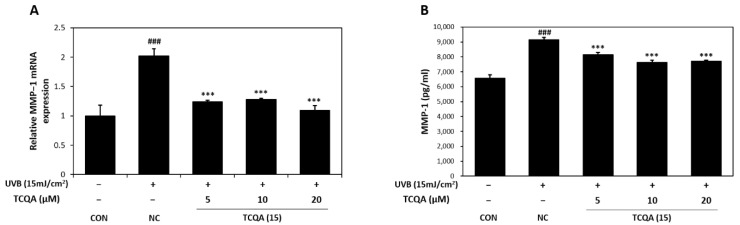
Effect of TCQA on MMP-1 expression. (**A**) MMP-1 mRNA expression was analyzed in HaCaT cells exposed to UVB by qRT-PCR and (**B**) the concentration of MMP-1 was assessed using ELISA. Data are expressed as mean ± SD (*n* = 2 to 3). ^###^ *p* < 0.001, versus CON group; *** *p* < 0.001, versus NC group. CON: untreated control group; NC: UVB-treated group; TCQA: TCQA-treated group.

The relative MMP-1 mRNA expression levels in HaCaT cells were measured by qRT-PCR. In comparison to the CON group, the NC group showed a two-fold increase in MMP-1 mRNA expression. However, the MMP-1 mRNA expression level after treatment with TCQA was significantly decreased by 1.7-fold at 5 μM, 1.5-fold at 10 μM, and 2-fold at 20 μM, compared to the NC group (Figure 7A). After UVB treatment, MMP-1 concentration measured by ELISA increased to 9142 pg/mL and TCQA treatment after UVB exposure reduced MMP-1 concentrations to 8152 pg/mL at 5 μM, 7619 pg/mL at 10 μM, and 7700 pg/mL at 20 μM (Figure 7B).

## 4. Discussion

Photoaging is a significant extrinsic factor that causes skin aging, and the increase in ROS caused by UV exposure upregulates the MAPK/NF-κB/AP-1 pathway and Nrf2–ARE signaling pathway and causes skin aging and wrinkling [46,47]. Recently, to prevent photoaging, natural products with antioxidant and anti-aging activities have been actively studied [25,26]. *N. peltata* is a perennial aquatic plant in the family Menyanthaceae and has been used in Traditional Chinese Medicine to treat heat strangury and polyuria [27,48]. Extracts of this plant have been reported to have anti-inflammatory and anti-tumor activities [30,48]. Furthermore, we previously reported on the anti-atopic and antioxidant activities of the EtOH extract of *N. peltata* [31]. In this study, we isolated and identified 15 phytochemicals, including a new oleanane-type triterpenoid saponin, 3-*O*-*β*-D-glucopyranosyl-oleanolic acid 28-*O*-*β*-D-glucuronopyranoside, in a methanolic extract of *N. peltata* roots and evaluated their protective effects on UVB-induced cellular injuries in HaCaT cells. Of these 15 compounds, α-spinasterol, ferulic acid, CQA, and TCQA markedly enhanced Nrf2–ARE luciferase activity; the antioxidant effects of α-spinasterol, ferulic acid, and CQA have been previously reported [43,44,45]. Therefore, we conducted further experiments on TCQA.

UV exposure causes oxidative stress and accelerates skin aging [49], and activation of the Nrf2 signaling pathway inhibits oxidative stress by regulating various antioxidant enzymes, including HO-1 [50]. These help to maintain skin homeostasis by inhibiting DNA damage, cell membrane destruction, and lipid damage [50]. HO-1 regulates oxidative stress and immune responses [51], and the Nrf2 pathway has been demonstrated to suppress ROS increases in UVB-exposed HaCaT cells and, thus, to alleviate apoptosis and reduce skin damage [52,53]. In the present study, TCQA exhibited strong antioxidant activity by upregulating Nrf2 and HO-1 expressions and reducing UVB-induced ROS levels in HaCaT cells. Furthermore, UVB exposure and oxidative stress induce the activation of NF-κB [54], which is involved in cellular senescence and apoptosis by causing protein damage [55]. They also induce the nuclear translocation of p65, which stimulates various inflammatory cells and the expressions of various inflammatory cytokines, like TNF-α and IFN-γ [56,57]. In addition, inflammation mediated by TNF-α enhances ROS production and increases NF-κB activation and the expressions of other cytokines [58]. In addition, activated NF-κB has been demonstrated to be involved in the synthesis of proteins that inhibit collagen production in dermal fibroblasts and HaCaT cells [59], disrupting skin homeostasis and inflammatory cycles, and causing rapid skin aging [60]. We also observed that TCQA suppressed NF-κB, p65, and IκBα transcript levels in UVB- and TNF-α/IFN-γ-induced HaCaT cells, which indicated that TCQA inhibits oxidative stress and inflammatory pathways.

Caffeoylquinic acid (CQA) derivatives are secondary plant metabolites, and many studies have reported their strong antioxidant and anti-inflammatory effects [61,62]. These bioactivities have been attributed to the presence of an *ortho*-hydroxy group and esterification groups on caffeic acid and quinic acid moieties [63,64]. According to the research on the different bioactivities of dicaffeoylquinic acid (DCQA) isomers, DCQAs (4,5- or 3,4-) with caffeoyl groups substituted at C-4 exhibit stronger physiological activities than 3,5-DCQA [65,66,67], which suggests the importance of the presence of the *cis*-caffeoyl group in DCQA [68]. Nevertheless, TCQA has been reported to have more potent pharmacological activities than DCQA. 3, 4, 5-TCQA has a stronger antiradical ability than DCQA [69], induces ATP synthesis [70], and effectively inhibits proinflammatory substances such as TNF-α [71]. This enhanced bioactivity has been attributed to increasing the steric hindrance of TCQA due to the presence of three caffeoyl groups [69,72]. Our in vitro results obtained using Nrf2–ARE reporter HaCaT cells confirmed that TCQA better inhibits Nrf2 activation compared with several CQAs and DCQAs.

MAPKs are composed of extracellular signal-regulated kinase (ERK), c-Jun N-terminal kinase (JNK), and p38 kinase [73]. They are involved in a variety of cellular activities, including cell proliferation, differentiation, and death [74]. UV-induced oxidative stress increases ROS levels and promotes the phosphorylation of ERK, JNK, and p38, thereby activating the MAPK signaling pathway [75,76]. In this study, TCQA significantly decreased the protein expressions of p-ERK, p-JNK, and p38 in UVB-irradiated HaCaT cells. Thus, our results suggest that TCQA inhibits UVB-induced MAPK activation. AP-1 is a transcription factor composed of c-Jun and c-Fos and is mediated by an upstream MAPK signaling pathway consisting of ERK, JNK, and p38 [77]. In other words, the expression of c-Fos is induced by ERK activation, and the expression of c-Jun is induced by JNK and p38 [78,79]. Furthermore, c-Fos and c-Jun combine to form either a heterodimer (Fos–Jun) or a homodimer (Jun–Jun), which regulate the transcriptions of numerous genes, including AP-1 [80]. Moreover, the activation of AP-1 transcription can induce ceramide production, leading to the overproduction of proinflammatory cytokines [81].

In the present study, TCQA significantly and dose-dependently reduced the protein expressions of phosphorylated c-Fos and c-Jun in UVB-irradiated HaCaT cells and significantly inhibited the dose-dependent upregulation of AP-1 induced by PMA exposure. The ROS-induced MAPK, AP-1, and NF-κB signaling pathways activate MMPs, which leads to MMP secretion from keratinocytes and fibroblasts, and accelerates protein and collagen degradation [82]. In particular, MMP-1 is a key marker of skin photoaging and causes collagen breakdown in the dermis [83], which contributes to the aging responses of dermal cells, such as dermal layer atrophy, skin wrinkling, and loss of elasticity [84]. TCQA inhibited the UVB-induced activation of MMP-1 at all concentrations examined. Taken together, we conclude that TCQA protects against photoaging and skin wrinkling by modulating MAPK/NF-κB/AP-1 signaling, reducing MMP-1 activity, and activating Nrf2 in UVB-damaged HaCaT cells.

## 5. Conclusions

Phytochemical investigation of the MeOH extract of *N. peltata* roots led to the isolation of 15 compounds (**1**–**15**). Among these compounds was a new oleanane-type triterpenoid saponin, 3-*O*-*β*-D-glucopyranosyl-oleanolic acid 28-*O*-*β*-D-glucuronopyranoside (peltatasaponin A), the chemical structure of which was characterized by 1D and 2D NMR spectroscopic data and HRESIMS, as well as chemical reaction. Among the isolates, TCQA effectively inhibited ROS production by inducing the expression of Nrf2/HO-1 antioxidant enzyme in HaCaT cells. In addition, treatment with TCQA significantly inhibited MMP-1 expression by suppressing MAPK, AP-1, and NF-kB pathways and their respective subunits in HaCaT cells exposed to UVB. Thus, our study supports the potential use of TCQA as an antioxidant and anti-wrinkle treatment.

## Figures and Tables

**Table 1:** ^1^H (400 MHz) and ^13^C NMR (100 MHz) data of compound **2** in DMSO-*d*_6_.

Compound 2					
Position	*δ*_H_ (Multi, *J* in Hz)	*δ*_C_	Position	*δ*_H_ (Multi, *J* in Hz)	*δ*_C_
Oleanolic acid			Glucose		
1	α: 0.88 (m); *β*: 1.48 (m)	38.6 CH_2_	1′	4.13 (d, 7.5)	105.8 CH
2	α: 1.50 (m); *β*: 1.90 (m)	25.9 CH_2_	2′	2.95 (m)	74.3 CH
3	3.01 (dd, 10.5, 3.5)	88.4 CH	3′	3.13 (m)	77.1 CH
4		39.2 C	4′	3.13 (m)	70.0 CH
5	0.71, overlap	55.5 CH	5′	3.13 (m)	78.2 CH
6	*α*: 1.46 (m); *β*: 1.30 (m)	18.2 CH_2_	6′	3.61 (dd, 11.0, 5.0); 3.43 (dd, 11.0, 5.5)	61.1 CH_2_
7	*α*: 1.36 (m); *β*: 1.21 (m)	32.7 CH_2_	2′-OH	4.87 (d, 4.0)	
8		39.6 C	3′-OH	4.92 (br. s)	
9	1.48 (m)	47.6 CH	4′-OH	5.04 (d, 4.0)	
10		36.7 C	6′-OH	4.45 (t, 5.5)	
11	1.79 (m)	23.4 CH_2_	Glucuronic acid		
12	5.16 (br. s)	122.2 CH_2_	1″	5.23 (d, 8.0)	94.6 CH
13		143.9 C	2″	3.11 (m)	72.8 CH
14		41.7 C	3″	3.21 (m)	77.1 CH
15	*α*: 0.95 (m); *β*: 1.72 (m)	27.6 CH_2_	4″	3.15 (m)	72.6 CH
16	*α*: 1.95 (m); *β*: 1.59 (m)	23.0 CH_2_	5″	3.28 (m)	74.6 CH
17		46.0 C	6″		174.3 C
18	2.73 (dd, 13.5, 3.0)	41.2 CH			
19		46.4 CH_2_			
20	*α*: 1.62 (m); *β*: 1.08 (m)	30.8 C			
21	*α*: 1.34 (m); *β*: 1.16 (m)	33.6 CH_2_			
22	1.51 (m)	32.1 CH_2_			
23-CH_3_	0.96 (s)	28.1 CH_3_			
24-CH_3_	0.74 (s)	17.0 CH_3_			
25-CH_3_	0.86 (s)	15.7 CH_3_			
26-CH_3_	0.67 (s)	17.1 CH_3_			
27-CH_3_	1.07 (s)	26.0 CH_3_			
28		175.7 C			
29-CH_3_	0.87 (s)	33.2 CH_3_			
30-CH_3_	0.86 (s)	23.8 CH_3_			

## Data Availability

Data are contained in the article.

## Supplementary Materials

The following are available online at https://www.mdpi.com/article/10.3390/antiox12101899/s1, Figure S1: The ^1^H NMR spectrum of compound **1**; Figures S2–S6: The ^1^H, ^13^C, ^1^H-^1^H COSY, HSQC, and HMBC NMR spectrum of compound **2**; Figures S7–S19: The ^1^H NMR spectrum of compound **3**–**15**.
