# Supplementary material for: Anti-Wrinkling Effect of 3,4,5-tri-O-caffeoylquinic Acid from the Roots of Nymphoides peltata through MAPK/AP-1, NF-κB, and Nrf2 Signaling in UVB-Irradiated HaCaT Cells
Source: Antioxidants (Basel). 2023 Oct 23;12(10):1899. doi: 10.3390/antiox12101899 (PMC10604296; doi:10.3390/antiox12101899)

Supplementary data

Anti-Wrinkling Effect of 3,4,5-tri-*O*-Caffeoylquinic Acid from the Roots of *Nymphoides peltata* through MAPK/AP-1, NF-κB, and Nrf2 Signaling in UVB-Irradiated HaCaT Cells

Tae-Young Kim [1,†], No-June Park [2,3,†], Beom-Geun Jo [1,†], Bum Soo Lee [4], Min-Ji Keem [1], Taek-Hwan Kwon [1], Ki Hyun Kim [4,*], Su-Nam Kim [2,3,*] and Min Hye Yang [1,*]

[1] Department of Pharmacy, College of Pharmacy and Research Institute for Drug Development, Pusan National University, Busan 46241, Republic of Korea
[2] Natural Products Research Institute, Korea Institute of Science and Technology, Gangneung 25451, Republic of Korea
[3] Division of Bio-Medical Science and Technology, KIST School, University of Science and Technology, Seoul 02792, Republic of Korea
[4] School of Pharmacy, Sungkyunkwan University, Suwon 16419, Republic of Korea
* Correspondence: khkim83@skku.edu (K.H.K.); snkim@kist.re.kr (S.-N.K.); mhyang@pusan.ac.kr (M.H.Y.); Tel.: +82-31-290-7700 (K.H.K.); +82-33-650-3503 (S.-N.K.); +82-51-510-2811 (M.H.Y.); Fax: +82-31-290-7730 (K.H.K.); +82-33-650-3419 (S.-N.K.); +82-51-513-6754 (M.H.Y.)
† These authors contributed equally to this work.

Supplementary Data Contents:

Figure S1. The ^{1}H NMR spectrum of compound **1** (Chloroform-*d*, 500 MHz) ……S1
Figure S2. The ^{1}H NMR spectrum of compound **2** (DMSO-d_6, 400 MHz)……S2
Figure S3. The ^{13}C NMR spectrum of compound **2** (DMSO-d_6, 100 MHz)……S3
Figure S4. The ^{1}H-^{1}H COSY spectrum of compound **2**……S4
Figure S5. The HSQC spectrum of compound **2**……S5
Figure S6. The HMBC spectrum of compound **2**……S6
Figure S7. The ^{1}H NMR spectrum of compound **3** (DMSO-d_6, 400 MHz)……S7
Figure S8. The ^{1}H NMR spectrum of compound **4** (DMSO-d_6, 400 MHz)……S8
Figure S9. The ^{1}H NMR spectrum of compound **5** (DMSO-d_6, 400 MHz)……S9
Figure S10. The ^{1}H NMR spectrum of compound **6** (DMSO-d_6, 400 MHz)……S10
Figure S11. The ^{1}H NMR spectrum of compound **7** (DMSO-d_6, 400 MHz)……S11
Figure S12 The ^{1}H NMR spectrum of compound **8** (DMSO-d_6, 400 MHz)……S12
Figure S13. The ^{1}H NMR spectrum of compound **9** (DMSO-d_6, 400 MHz)……S13
Figure S14. The ^{1}H NMR spectrum of compound **10** (DMSO-d_6, 400 MHz)……S14
Figure S15. The ^{1}H NMR spectrum of compound **11** (DMSO-d_6, 400 MHz)……S15
Figure S16. The ^{1}H NMR spectrum of compound **12** (DMSO-d_6, 400 MHz)……S16
Figure S17. The ^{1}H NMR spectrum of compound **13** (DMSO-d_6, 400 MHz)……S17
Figure S18. The ^{1}H NMR spectrum of compound **14** (DMSO-d_6, 400 MHz)……S18
Figure S19. The ^{1}H NMR spectrum of compound **15** (DMSO-d_6, 400 MHz)……S19

Figure S1. The ^{1}H NMR spectrum of compound **1** (Chloroform-*d*, 500 MHz)

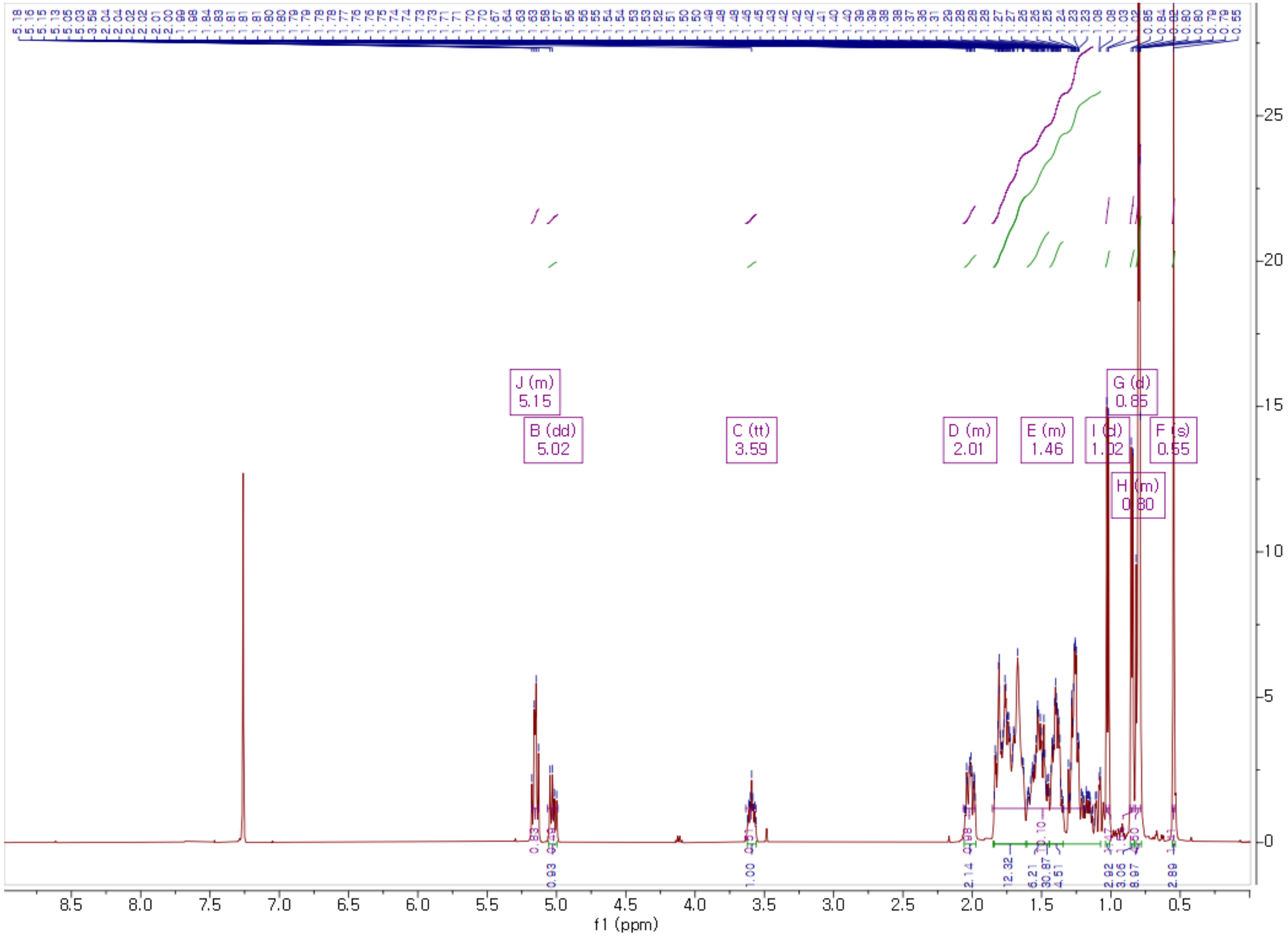

Figure S2. The ^{1}H NMR spectrum of compound **2** (DMSO-d_6, 400 MHz)

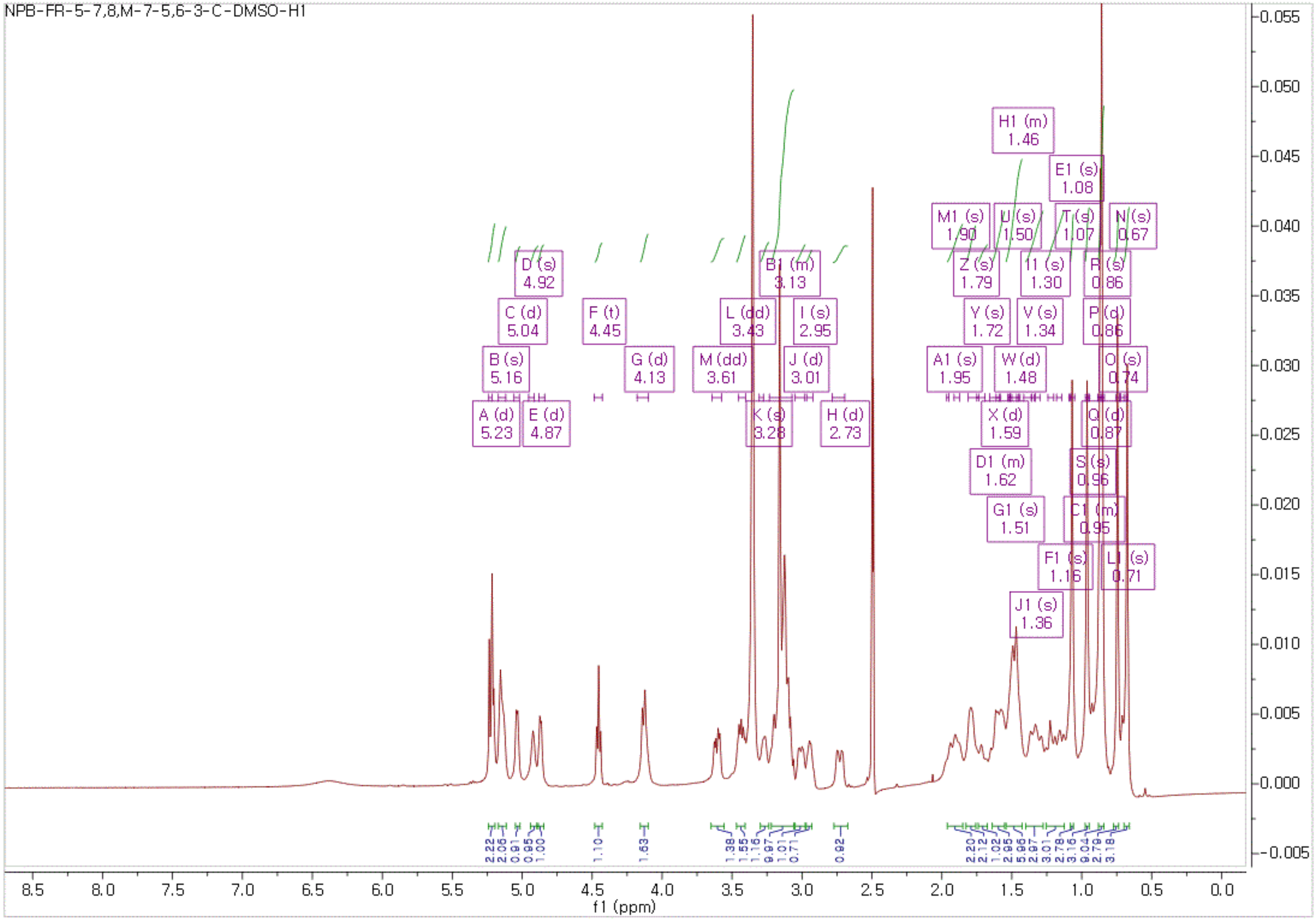

Figure S3. The ^{13}C NMR spectrum of compound **2** (DMSO-d_6, 100 MHz)

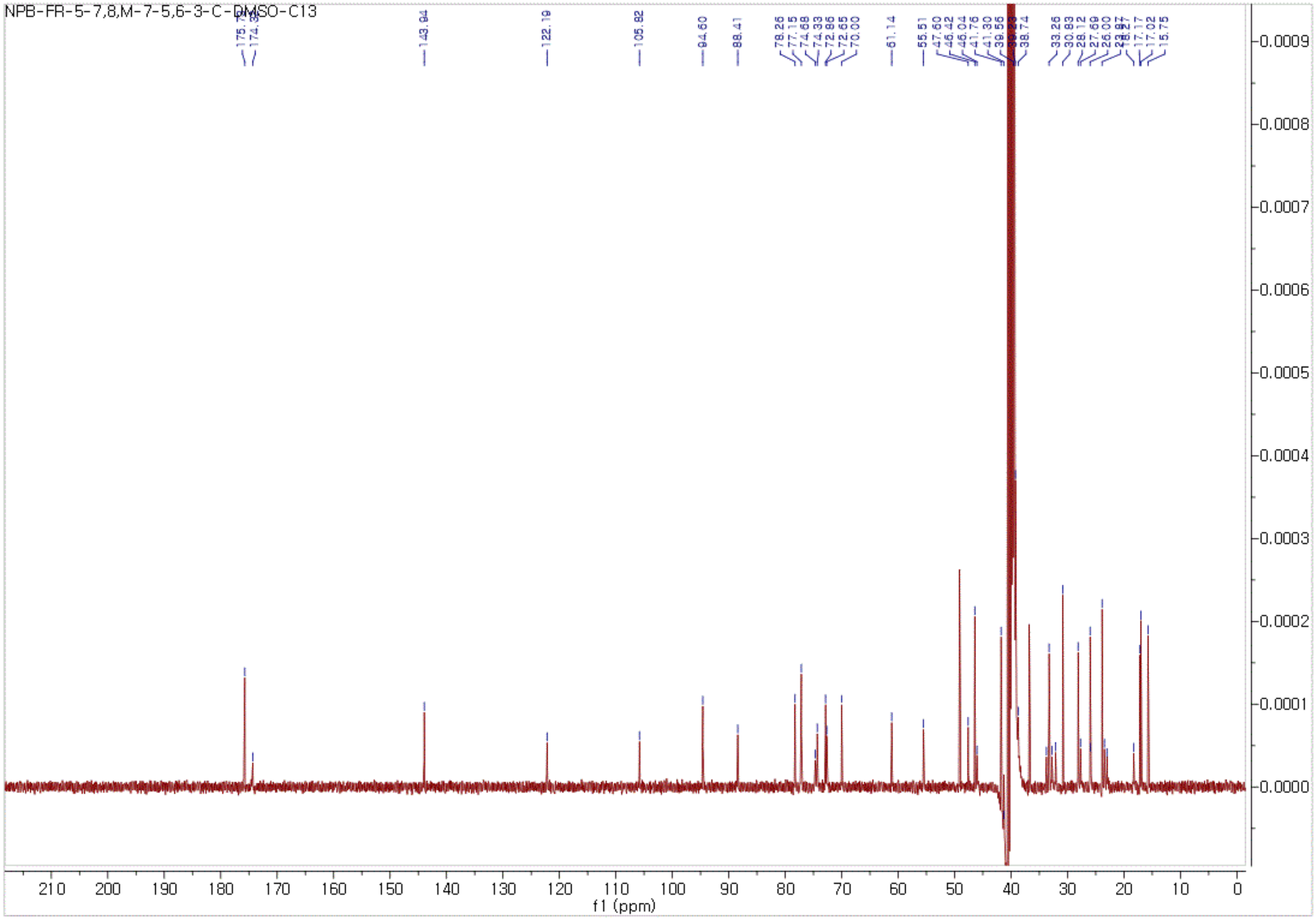

Figure S4. The ^{1}H-^{1}H COSY spectrum of compound **2**

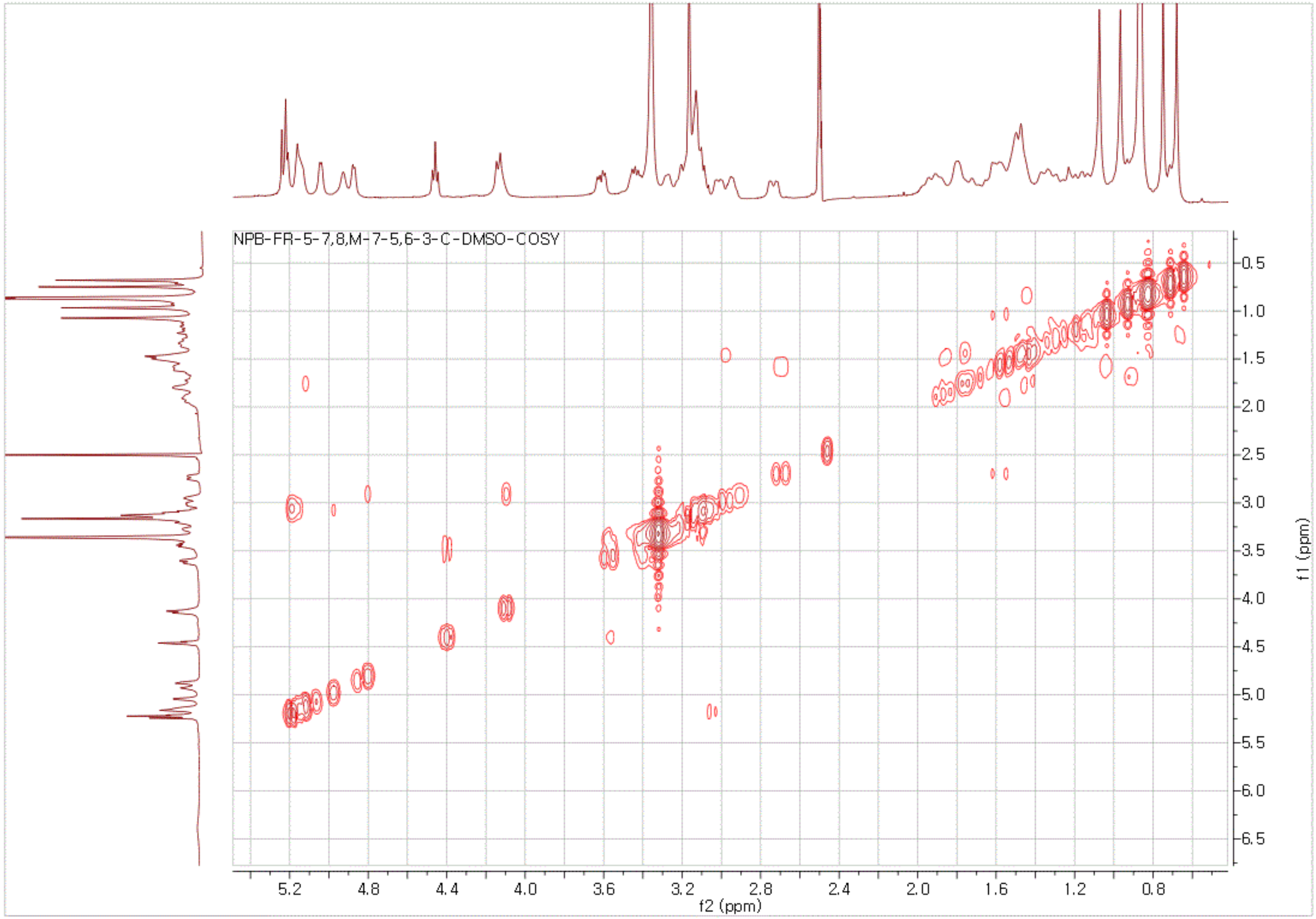

Figure S5. The HSQC spectrum of compound **2**

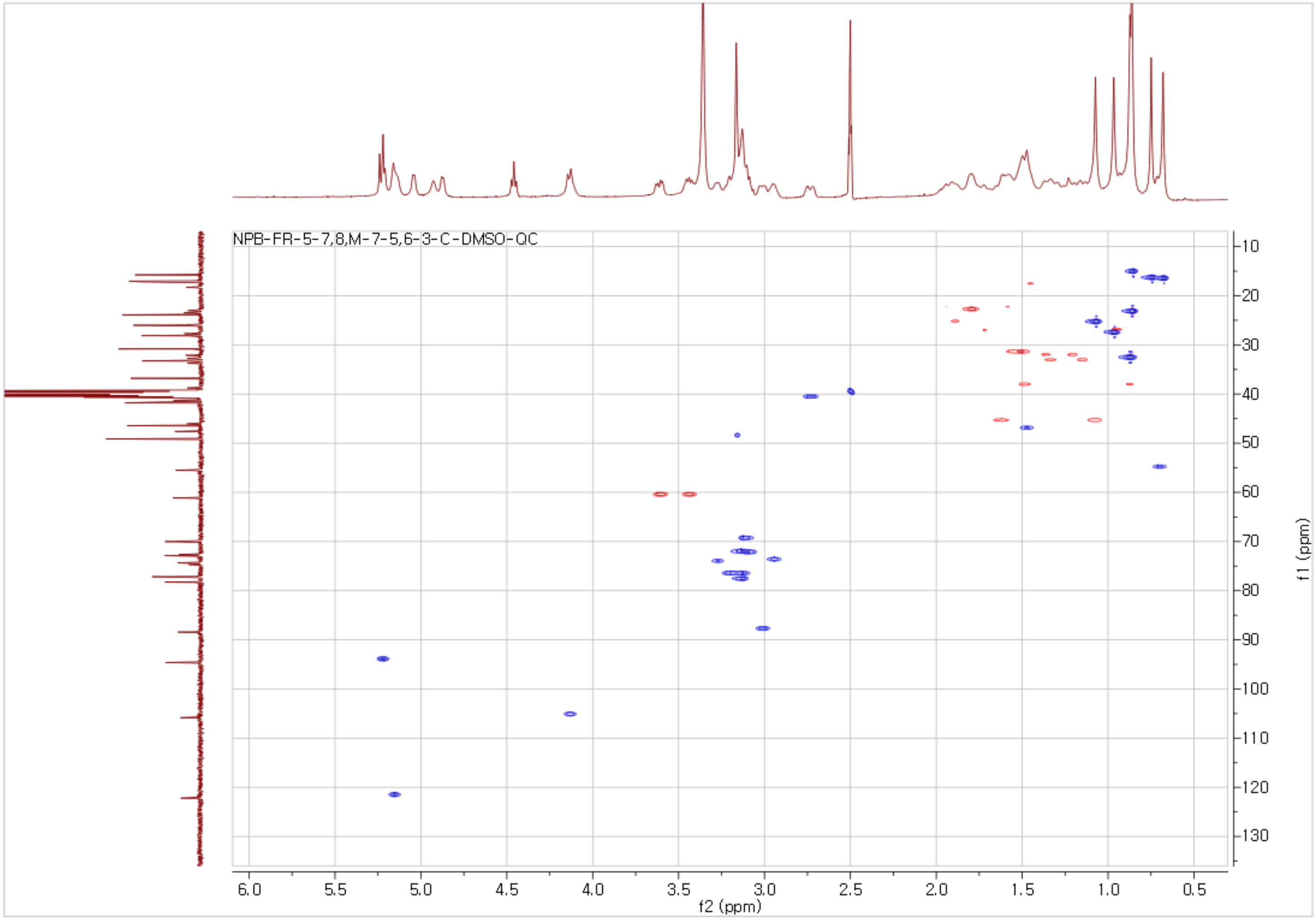

Figure S6. The HMBC spectrum of compound **2**

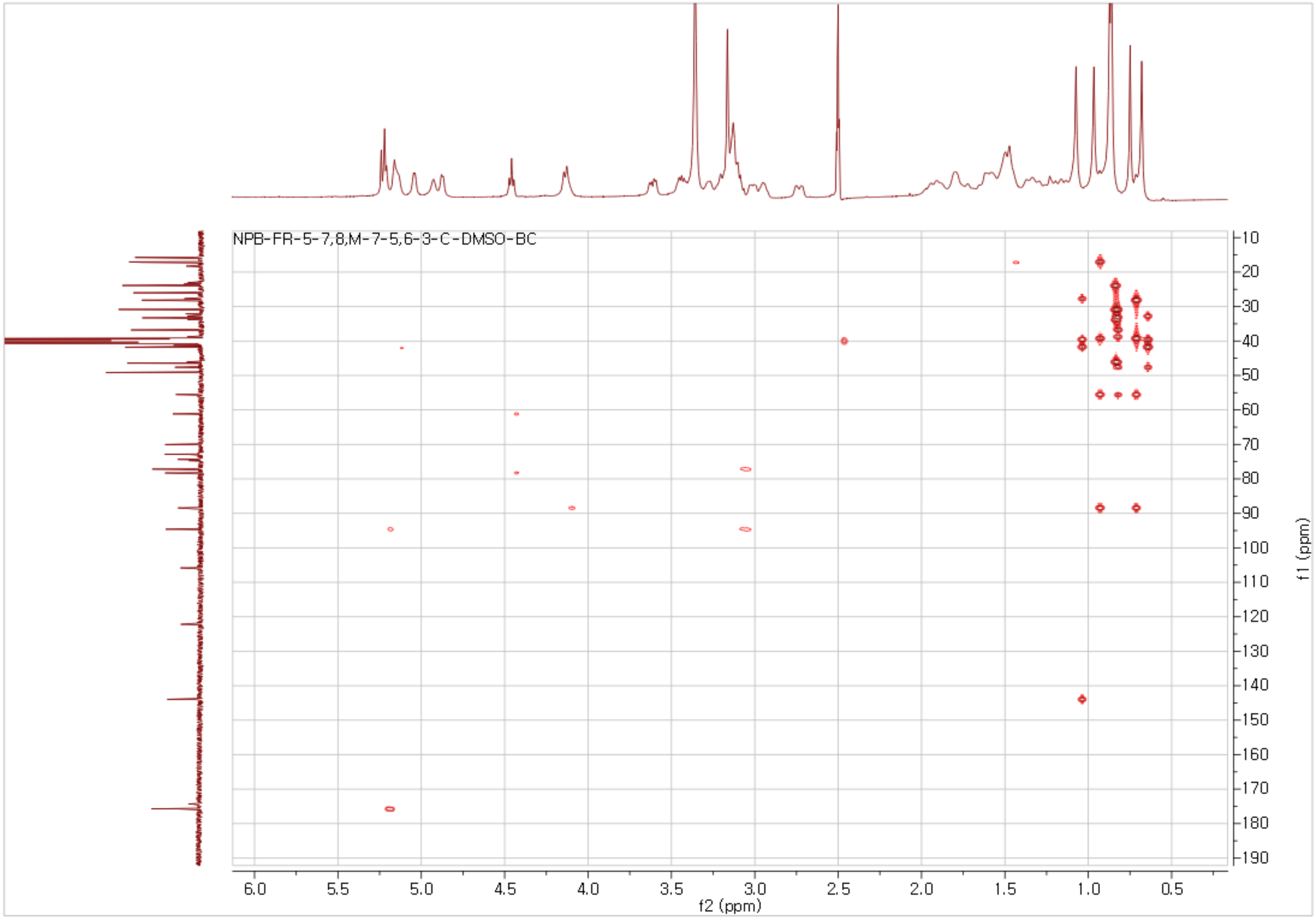

Figure S7. The ^{1}H NMR spectrum of compound **3** (DMSO-d_6, 400 MHz)

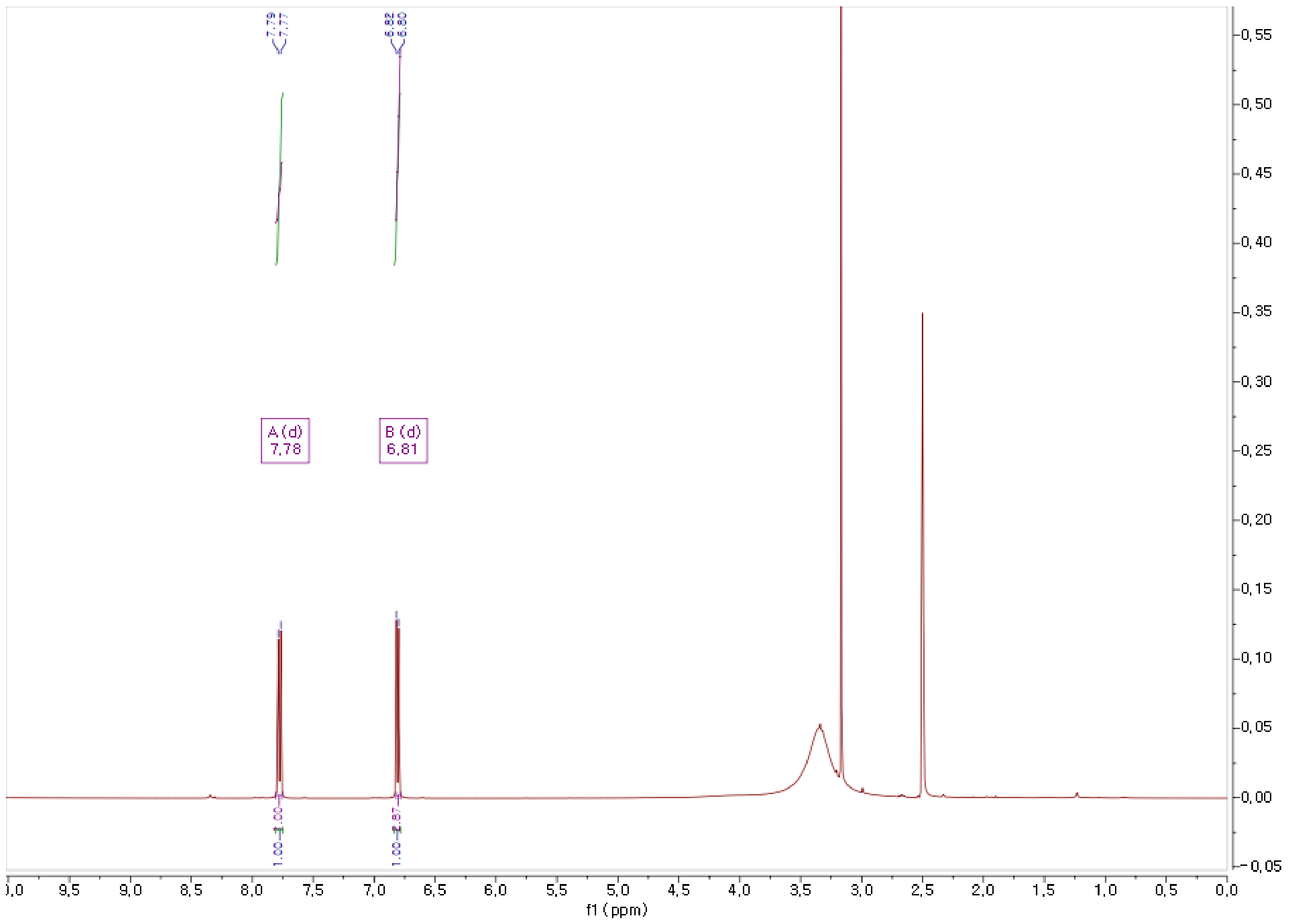

Figure S8. The ^{1}H NMR spectrum of compound **4** (DMSO-d_6, 400 MHz)

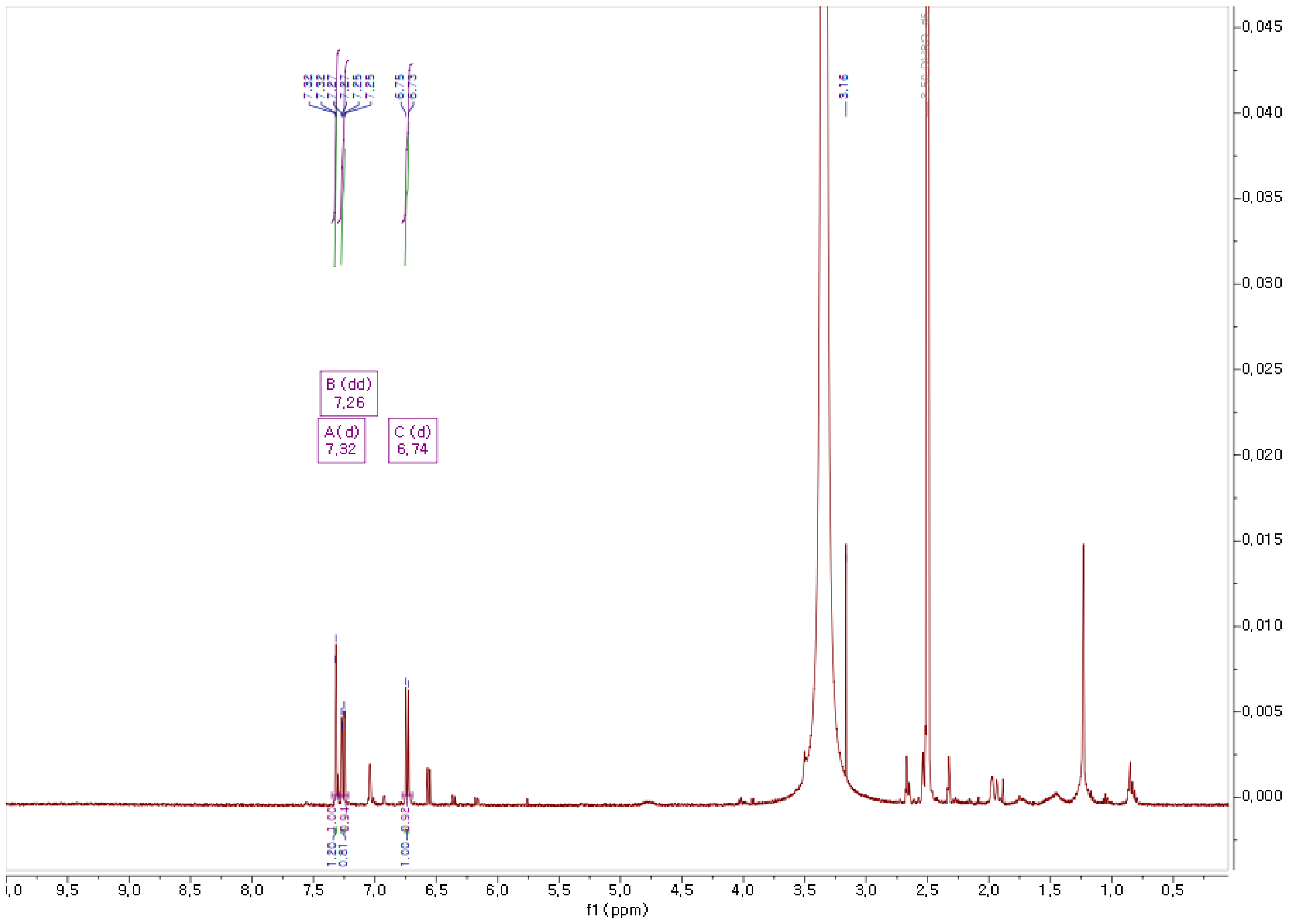

Figure S9. The ^{1}H NMR spectrum of compound **5** (DMSO-d_6, 400 MHz)

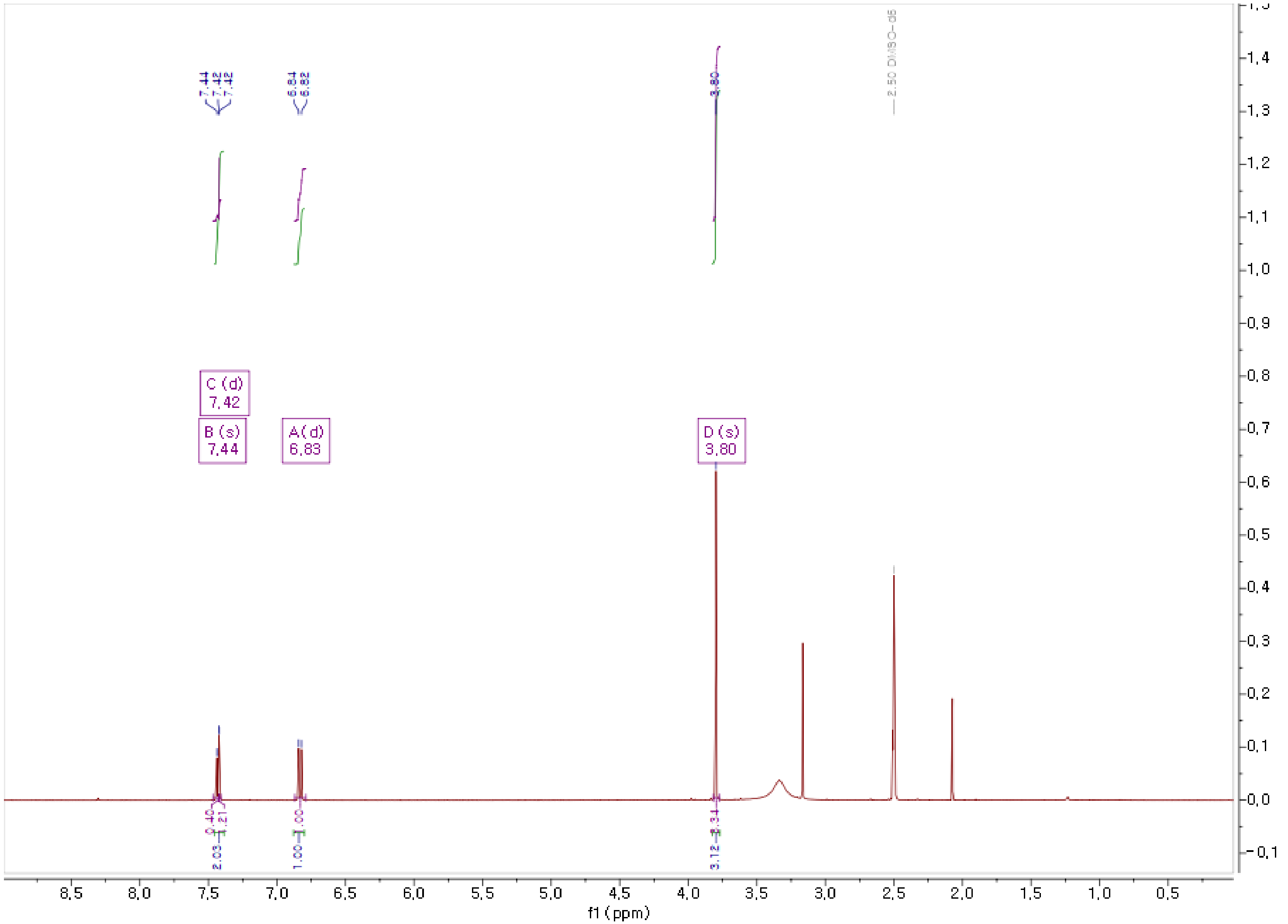

Figure S10. The ^{1}H NMR spectrum of compound **6** (DMSO-d_6, 400 MHz)

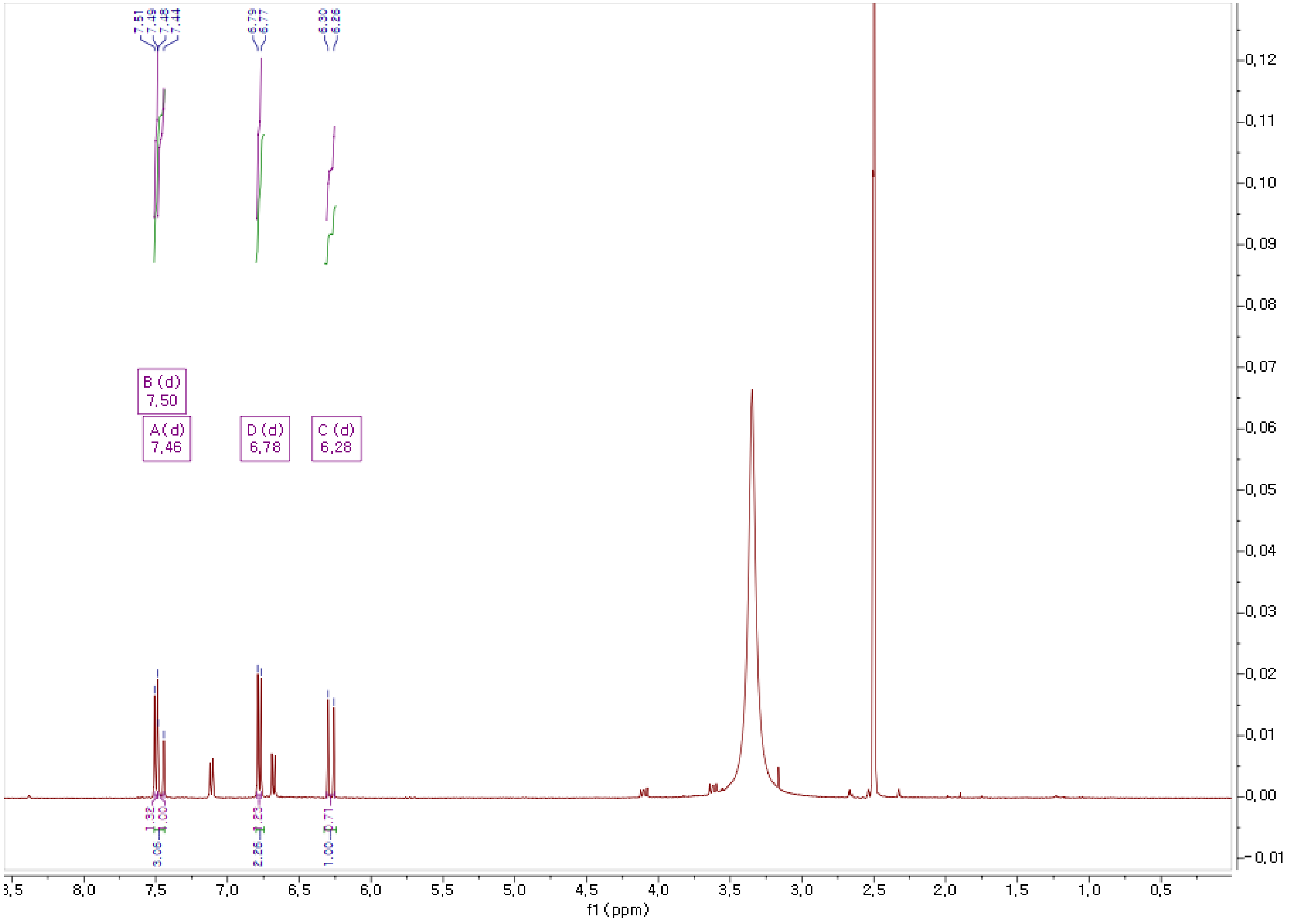

Figure S11. The ^{1}H NMR spectrum of compound **7** (DMSO-d_6, 400 MHz)

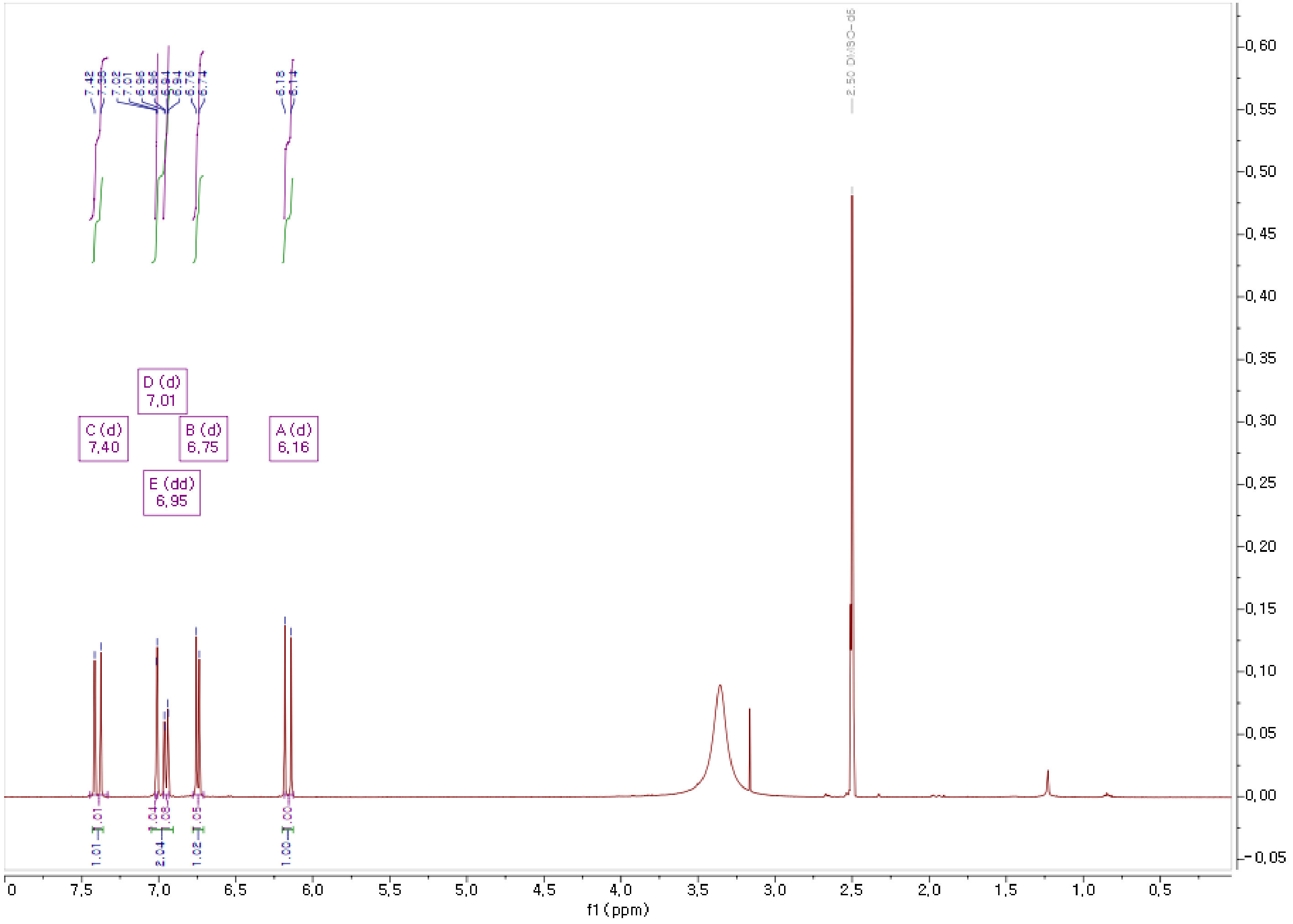

Figure S12. The ^{1}H NMR spectrum of compound **8** (DMSO-d_6, 400 MHz)

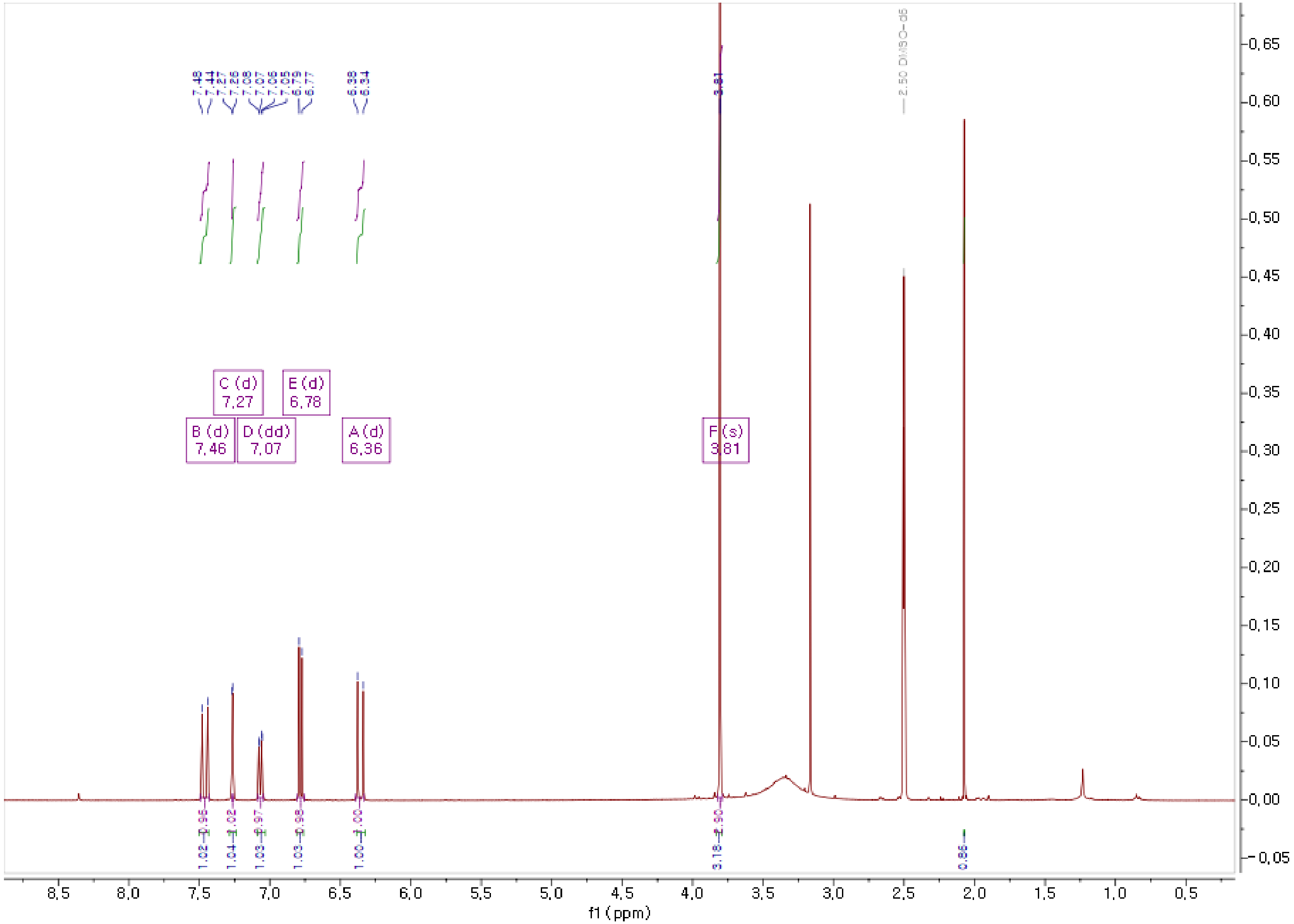

Figure S13. The ^{1}H NMR spectrum of compound **9** (DMSO-d_6, 400 MHz)

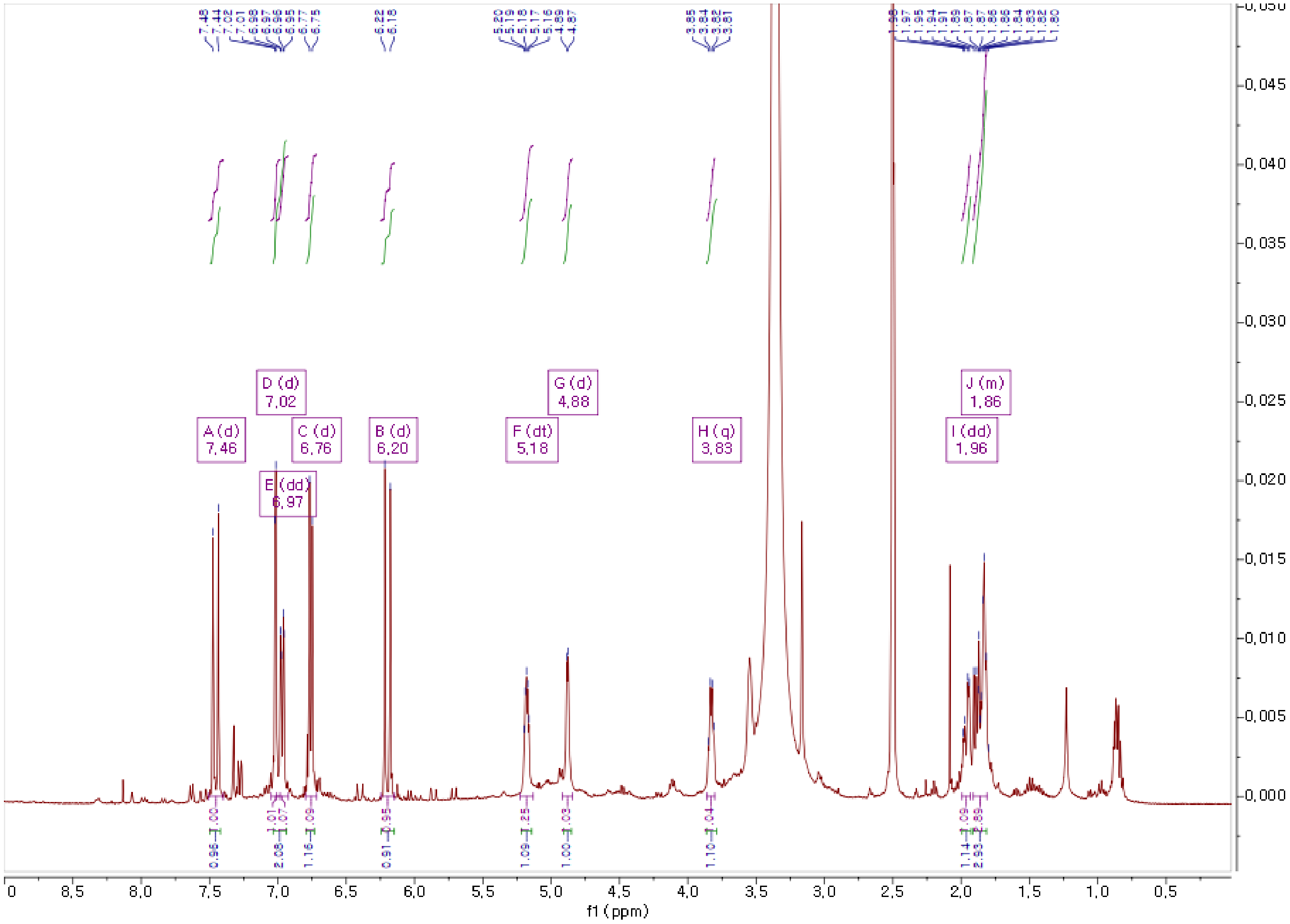

Figure S14. The ^{1}H NMR spectrum of compound **10** (DMSO-d_6, 400 MHz)

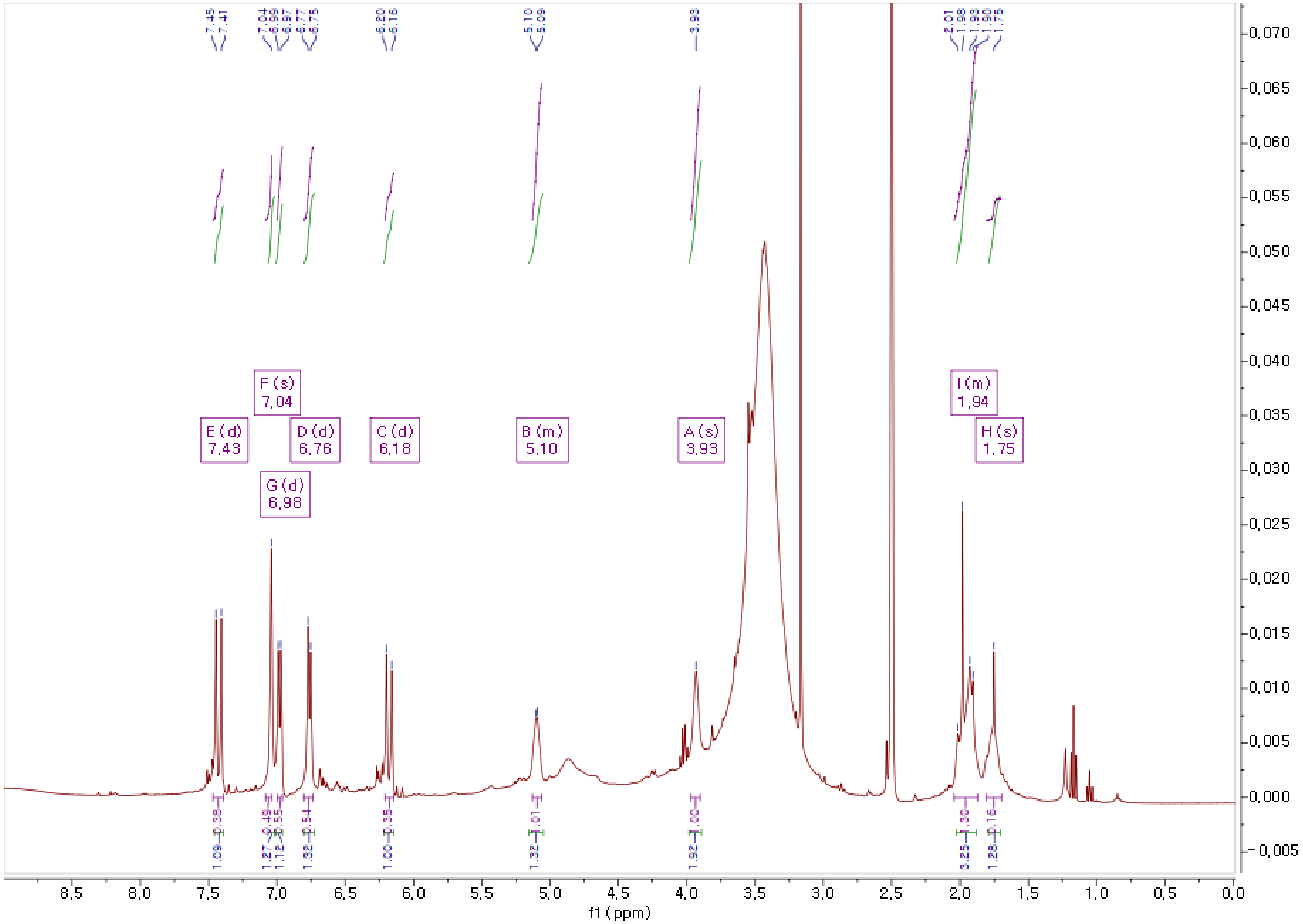

Figure S15. The ^{1}H NMR spectrum of compound **11** (DMSO-d_6, 400 MHz)

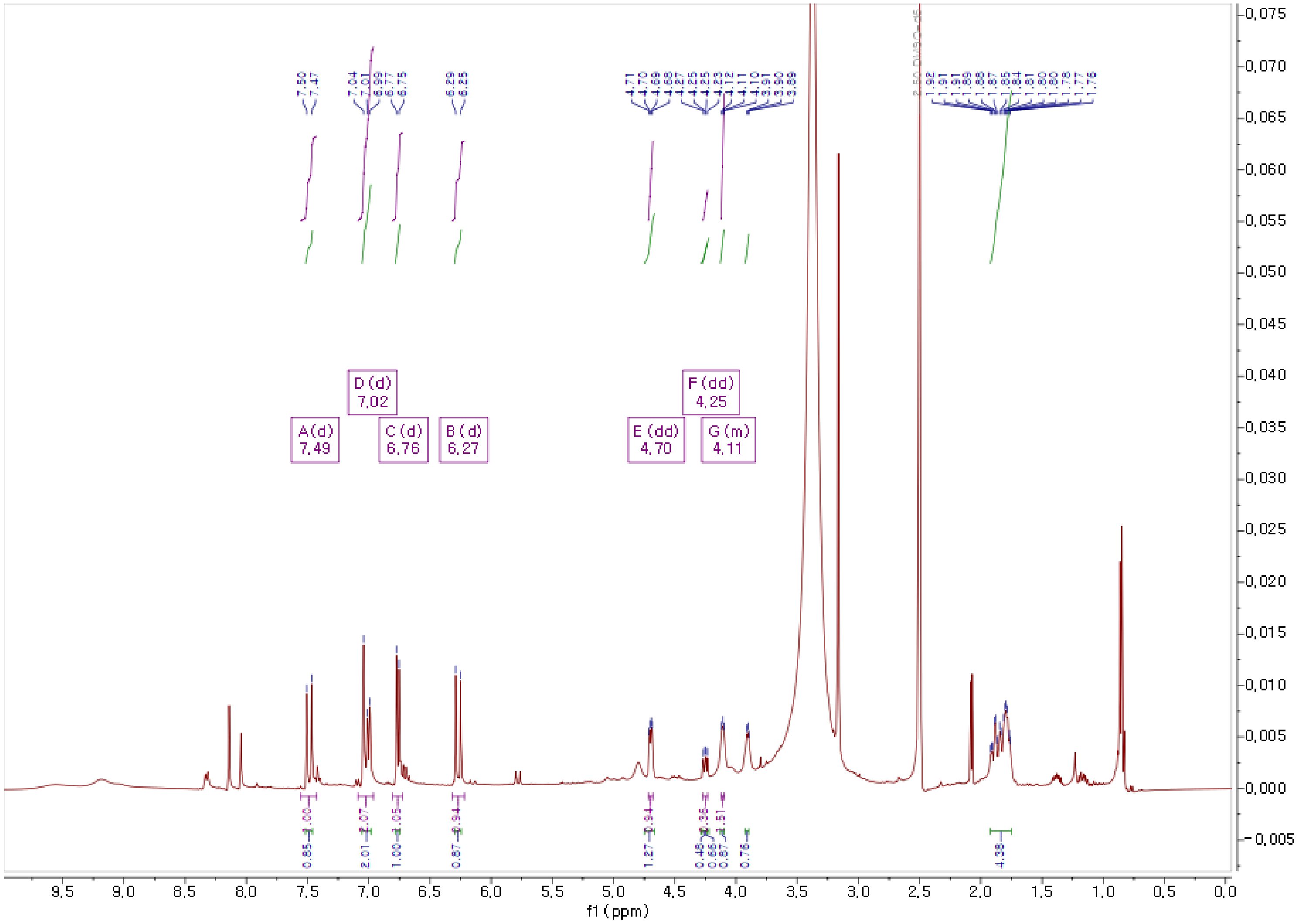

Figure S16. The ^{1}H NMR spectrum of compound **12** (DMSO-d_6, 400 MHz)

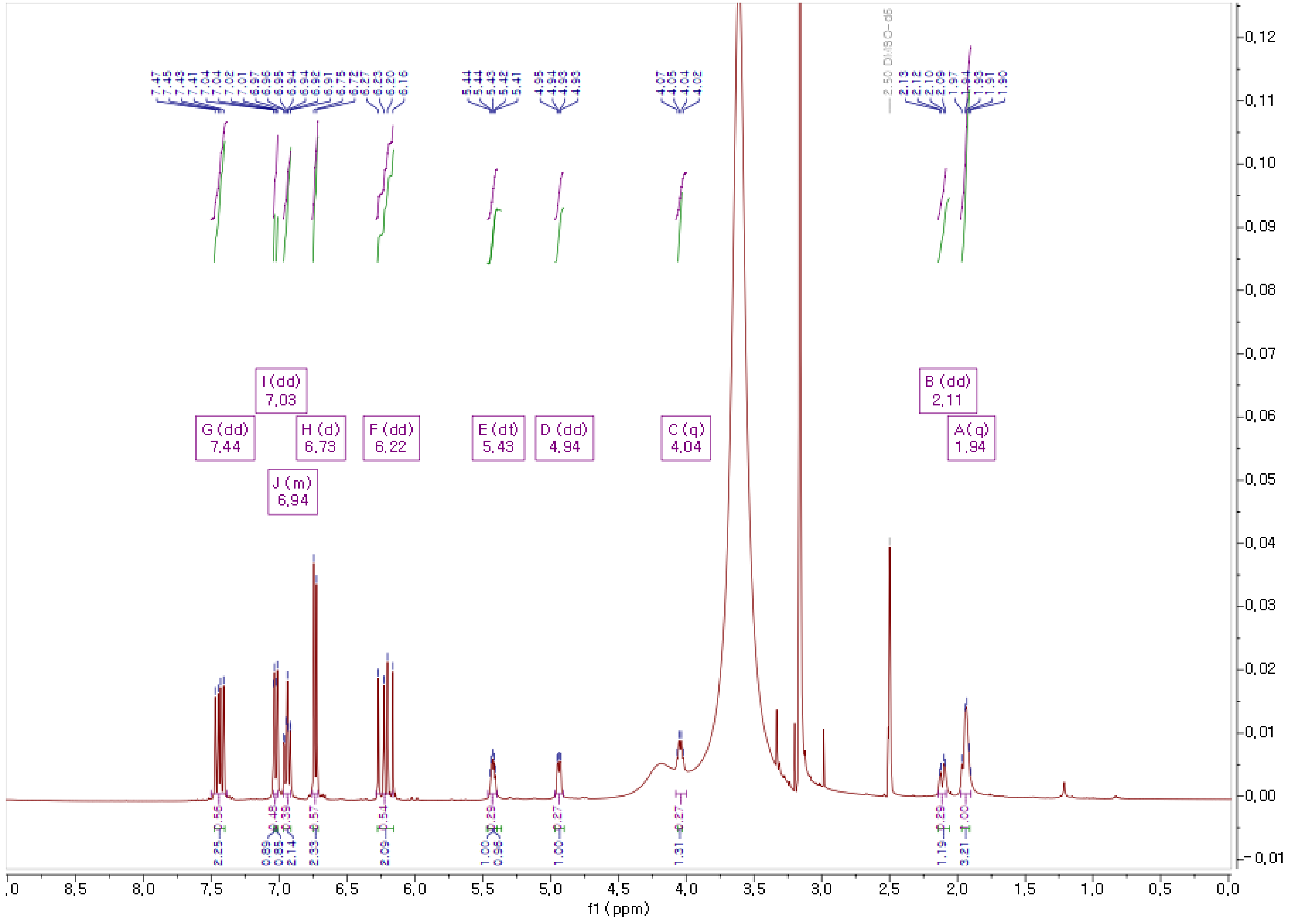

Figure S17. The ^{1}H NMR spectrum of compound **13** (DMSO-d_6, 400 MHz)

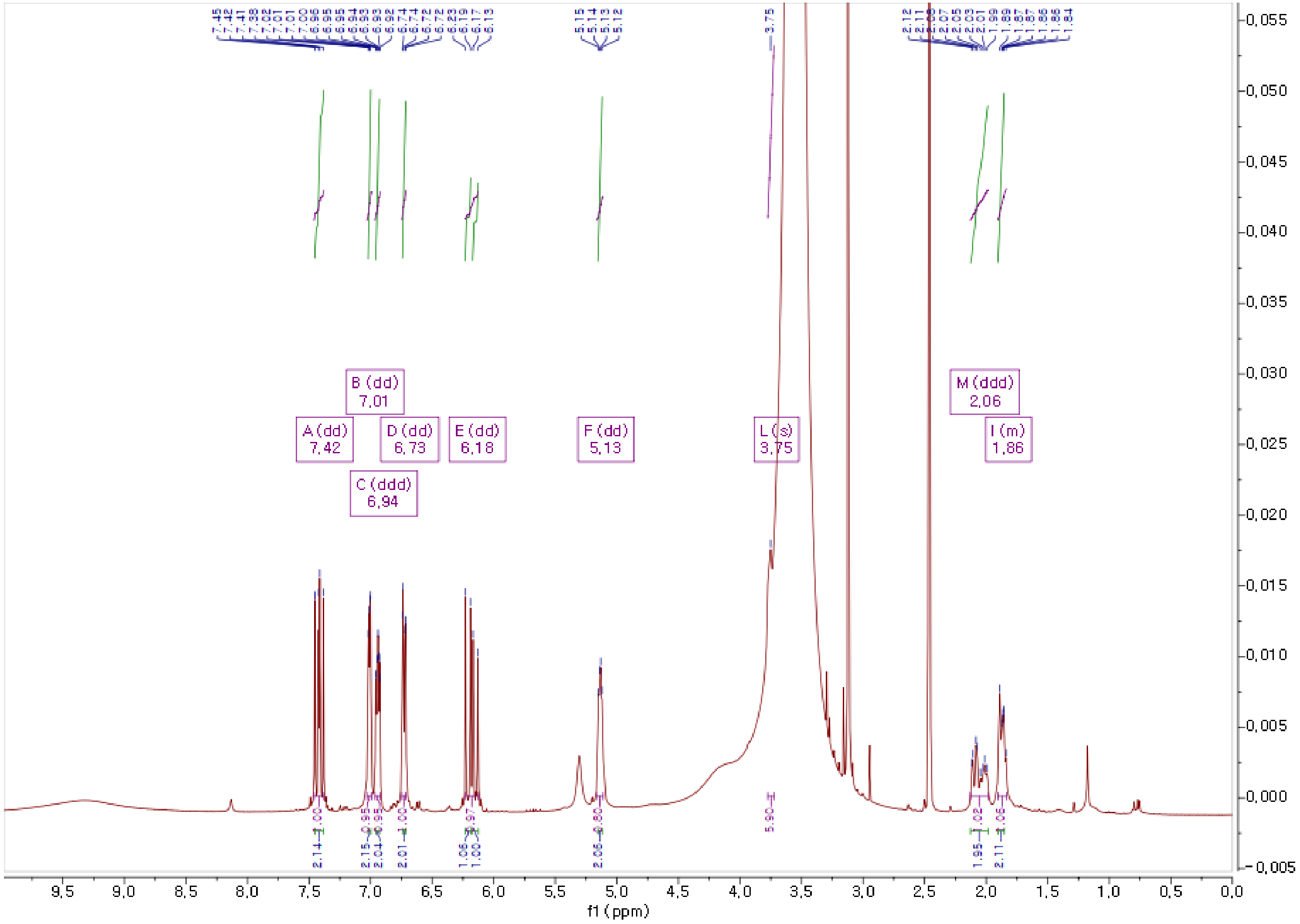

Figure S18. The ^{1}H NMR spectrum of compound **14** (DMSO-d_6, 400 MHz)

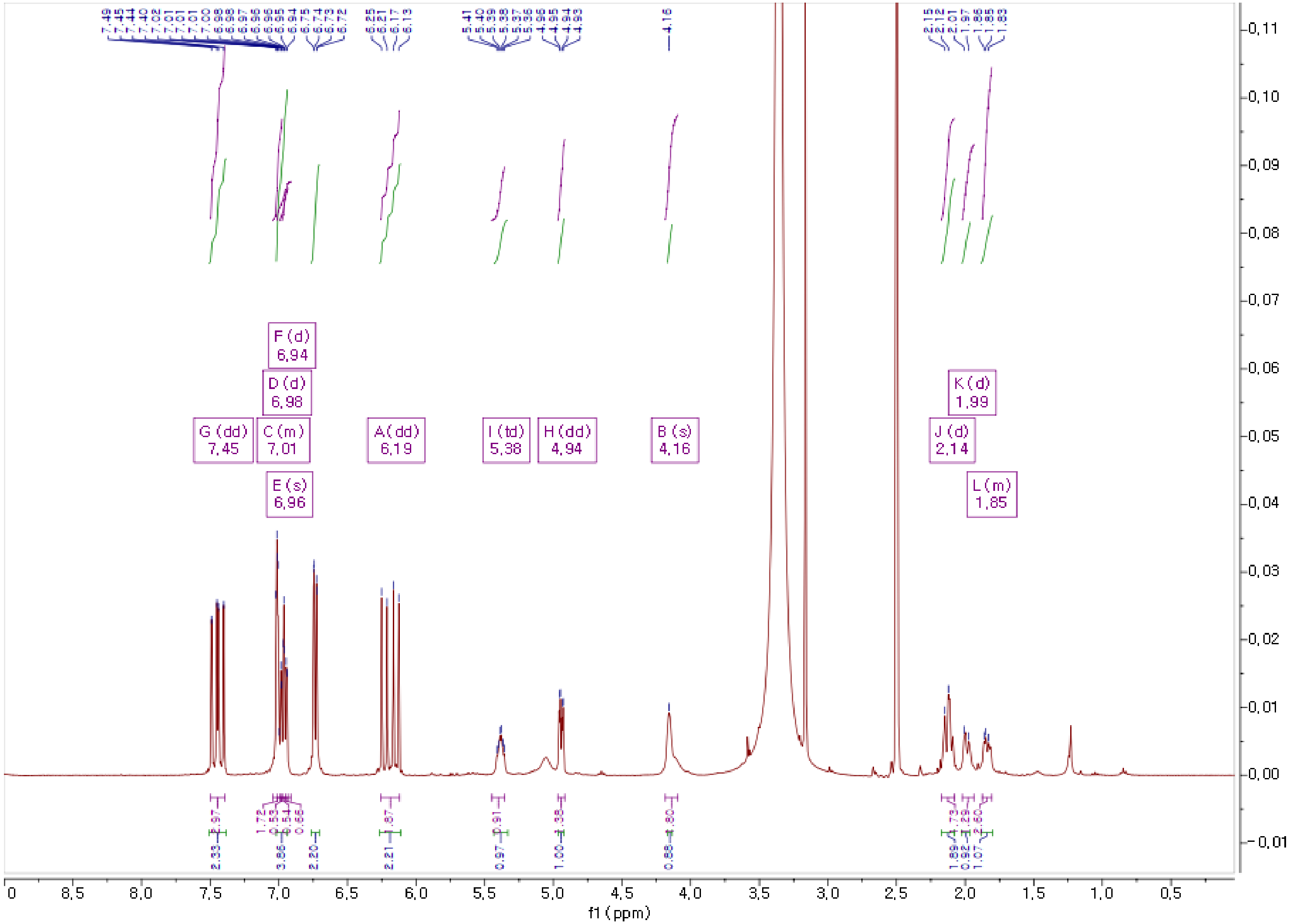

Figure S19. The ^{1}H NMR spectrum of compound **15** (DMSO-d_6, 400 MHz)

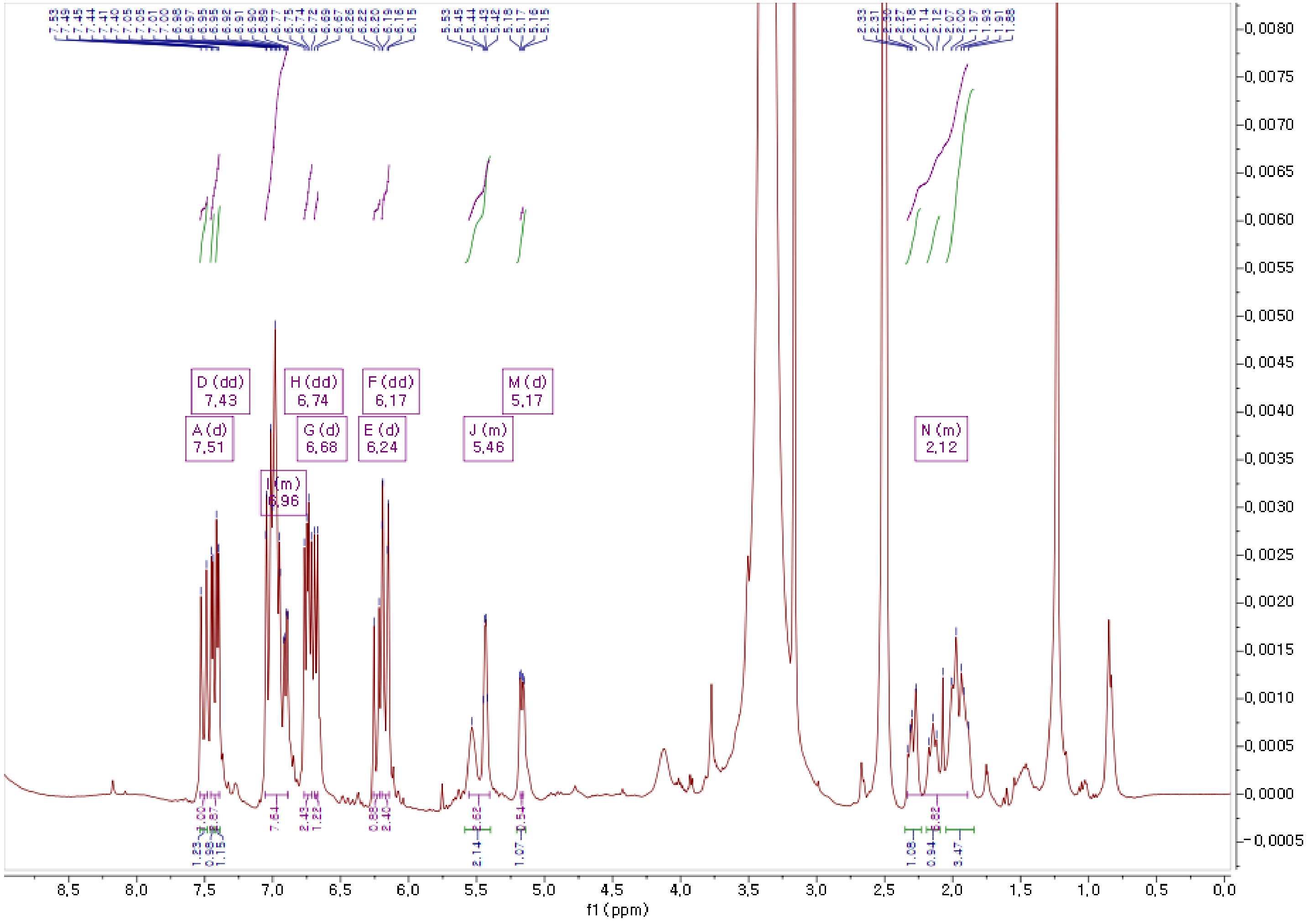

Supplement: Supplementary file 1 [file antioxidants-12-01899-s001.zip › antioxidants-2650119-supplementary.pdf]
